# Transcriptional analysis of phloem-associated cells of potato

**DOI:** 10.1186/s12864-015-1844-2

**Published:** 2015-09-03

**Authors:** Tian Lin, Coralie C. Lashbrook, Sung Ki Cho, Nathaniel M. Butler, Pooja Sharma, Usha Muppirala, Andrew J. Severin, David J. Hannapel

**Affiliations:** Plant Biology, Iowa State University, 253 Horticulture Hall, Ames, IA 50011-1100 USA; Department of Plant Breeding, Genetics, and Biotechnology, Michigan State University, East Lansing, MI 48824 USA; Office of Biotechnology, Iowa State University, Ames, IA 50011-3210 USA

**Keywords:** Laser capture microdissection, Mobile RNA, Photoperiod, Polypyrimidine tract binding, RNA-binding proteins, Sieve elements, Signal, Vascular biology

## Abstract

**Background:**

Numerous signal molecules, including proteins and mRNAs, are transported through the architecture of plants via the vascular system. As the connection between leaves and other organs, the petiole and stem are especially important in their transport function, which is carried out by the phloem and xylem, especially by the sieve elements in the phloem system. The phloem is an important conduit for transporting photosynthate and signal molecules like metabolites, proteins, small RNAs, and full-length mRNAs. Phloem sap has been used as an unadulterated source to profile phloem proteins and RNAs, but unfortunately, pure phloem sap cannot be obtained in most plant species.

**Results:**

Here we make use of laser capture microdissection (LCM) and RNA-seq for an in-depth transcriptional profile of phloem-associated cells of both petioles and stems of potato. To expedite our analysis, we have taken advantage of the potato genome that has recently been fully sequenced and annotated. Out of the 27 k transcripts assembled that we identified, approximately 15 k were present in phloem-associated cells of petiole and stem with greater than ten reads. Among these genes, roughly 10 k are affected by photoperiod. Several RNAs from this day length-regulated group are also abundant in phloem cells of petioles and encode for proteins involved in signaling or transcriptional control. Approximately 22 % of the transcripts in phloem cells contained at least one binding motif for Pumilio, Nova, or polypyrimidine tract-binding proteins in their downstream sequences. Highlighting the predominance of binding processes identified in the gene ontology analysis of active genes from phloem cells, 78 % of the 464 RNA-binding proteins present in the potato genome were detected in our phloem transcriptome.

**Conclusions:**

As a reasonable alternative when phloem sap collection is not possible, LCM can be used to isolate RNA from specific cell types, and along with RNA-seq, provides practical access to expression profiles of phloem tissue. The combination of these techniques provides a useful approach to the study of phloem and a comprehensive picture of the mechanisms associated with long-distance signaling. The data presented here provide valuable insights into potentially novel phloem-mobile mRNAs and phloem-associated RNA-binding proteins.

**Electronic supplementary material:**

The online version of this article (doi:10.1186/s12864-015-1844-2) contains supplementary material, which is available to authorized users.

## Background

Plants are sessile organisms and unlike animals have no neural network or circulatory system. Phloem and xylem are the main tissues that facilitate nutrient and signal transport in the whole-plant body. With the evolution in size and complexity, the need for an efficient long-distance transport system has steadily increased over time for land plants [1]. The result of these changes has led to the development of more specialized and complicated cell types in both the phloem and xylem. Xylem is composed of parenchyma cells, fibers and long tracheary elements that transport water and soluble mineral ions from the root to other organs. Tracheary elements and fibers are enucleate, non-living cells that maintain only a cell wall. In comparison, phloem is composed of living cell types, including sieve elements, parenchyma cells, and supportive cells, such as fibers and sclereids. Parenchyma cells include both specialized companion cells and unspecialized phloem parenchyma cells. Sieve elements lose most of their organelles and are enucleate. All their metabolic functions are carried out by the companion cells but profiles of phloem proteins suggest that translation may occur within the sieve element system [2]. RNAs are transcribed and translated in companion cells and small RNAs, mRNAs and proteins are then actively transported into sieve elements through the plasmodesmata [3].

Phloem is the conduit for transport of photosynthates, mainly sucrose, from leaf to sink tissues. Signal molecules also take advantage of this information highway to communicate between different organs. These molecules can be hormones [4], small RNAs [5], full-length mRNAs [6–12] and proteins like FT [13, 14]. From numerous studies of phloem-mobile signals, it is clear that these molecules can be delivered in either an acropetal or basipetal direction. Two examples illustrate how such phloem-mobile signals regulate development [15]. Under photoperiodic conditions inductive for flowering, FLOWERING LOCUS T (FT) is expressed in the leaf and transported in protein form through the sieve element system to the shoot apex where, in conjunction with FLOWERING LOCUS D (FD), it activates the floral pathway [16]. Several studies have identified FT in the shoot apex or phloem exudate of plants induced for flowering [13, 17, 18]. In this system, FD provides spatial control of flowering and FT provides temporal control. As another example, using heterografting experiments, full-length *StBEL5* mRNA of potato was verified to move in a downward direction from leaf to stolon and root [9, 19]. This long-distance phloem transport of *StBEL5* is enhanced under short days and controlled by untranslated regions of the transcript. Movement of *StBEL5* mRNA was correlated with increased tuber yields and root growth [9, 19]. Both of these long-distance signaling systems utilize photoperiodic cues to activate movement of the developmental signal from source to sink organs.

The mechanism of non-cell autonomous movement and its regulation are still unclear, but RNA-binding proteins (RBP) identified from phloem sap of pumpkin bind to mobile mRNAs to regulate their movement [2, 20]. A polypyrimidine tract-binding (PTB) protein of pumpkin (RBP50) was identified as the core protein of a RNA/protein complex that transports RNA. Further evidence suggests that similar RBPs in potato function to facilitate both stability and long-distance transport of select mobile RNAs [21]. Transcription of these RBPs was observed in companion cells of the phloem [20]. To elucidate the potential for long-distance signaling through the sieve element system, several profiles of phloem proteins and RNAs have been undertaken. Analysis of the proteome of phloem sap of pumpkin revealed over 1200 proteins present in the sieve element system [2]. Through both phloem cell microdissection and analysis of phloem sap, we now know that the transcriptome of phloem includes thousands of full-length mRNAs with a diverse range of potential functions [22, 23]. Phloem sap, in particular, has been used as an efficient source to study uncontaminated phloem proteins and RNAs [2, 20, 24]. Results from the most widely used model system for phloem sap analysis, the cucurbits, have been compromised, however, due to the existence of two separate phloem sources each with unique protein and RNA sets [25]. In most plant species pure phloem sap cannot be obtained. As a reasonable alternative, laser capture microdissection (LCM) makes it possible to isolate RNA from specific cell types and provides practical access to expression profiles of phloem tissue [26–29]. In previous studies, transcripts of seven of the *StBEL* genes of potato, including the mobile mRNA, *StBEL5*, were identified in RNA extracted from phloem cells using LCM/RT-PCR [30]. Combining LCM and RNA-seq has proven to be an invaluable tool for profiling high-resolution transcription in specific cells [28, 31–34]. Here we make use of LCM and RNA-seq for an in-depth transcriptional profile of phloem-associated cells of both petioles and stems of potato. The combination of these techniques has provided a valuable approach to the study of phloem tissue and a comprehensive snapshot into the mechanisms associated with long-distance signaling.

## Results

### Analysis of a LCM phloem transcriptome

**Fig. 1:**
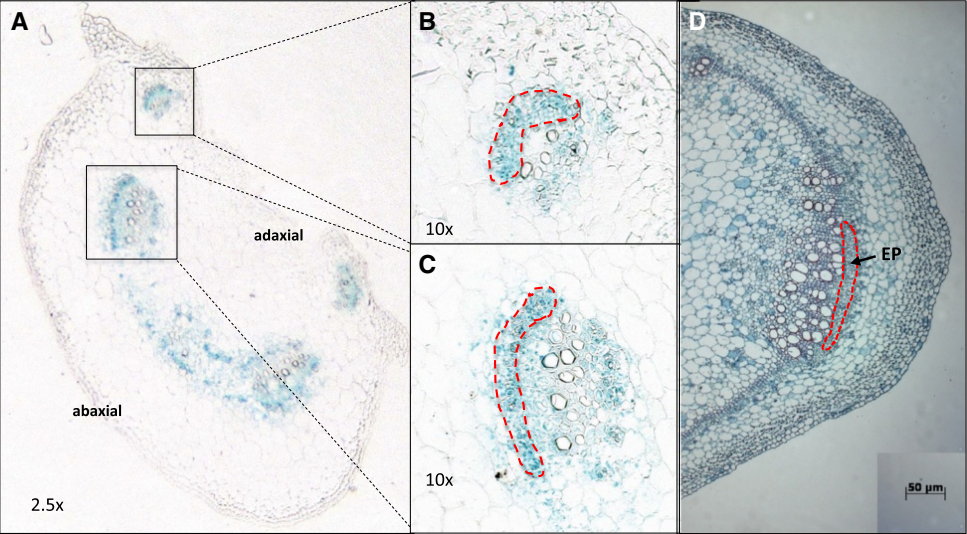
Transverse section of potato petiole and stem to show phloem tissue collected in LCM with phloem specific marker *StPTB1*. Localization of GUS activity within petioles (**a**-**c**) and stems (**d**) of plants that contained the *StPTB1* promoter driving GUS expression [35]. Petiole and stem internodes of tuberizing 4-week old soil grown plants were embedded in paraffin for histochemical detection of GUS activity within tissues of the petiole and stem. Panel (**a**) is a transverse section of *StPTB1prom* activity with a higher magnification image of a vascular bundle (*boxed area*) in (**b**-**c**) showing abaxial-side phloem cells. Panel (**d**) is a transverse section of *StPTB1prom* activity in the vascular bundle of a stem showing external (EP) phloem tissues

To gain insight into the function of the numerous genes actively involved in transport and signaling throughout the phloem system, transcriptomes of phloem-associated cells (PAC) were profiled from both the petiole and the lower stem of short day-grown potato plants. The petiole was selected because of its proximity to the leaf, an important source of a wide range of light-activated and photosynthate-related signals. The lower stem was selected because of its proximity to the strong tuber sink. RNA was isolated from phloem tissue samples dissected from paraffin-embedded petiole (Short day [SD] Petiole-phloem) and stem sections (SD Stem-phloem) using the LCM method [30]. Because of our interest in the short day (SD) activated process of tuber formation in potato, both samples come from SD-grown plants. The sample collected by LCM contains not only sieve elements, but also the companion cells, phloem parenchyma cells and other cells associated with the phloem. Making use of a phloem-specific marker, *StPTB1* [35], phloem cells that were harvested can be observed in scattered bundles in the petiole (Fig. 1a-c) and in outer regions of discrete vascular bundles in the stem (Fig. 1d). Based on this morphology, the transcriptome profiled from the LCM-derived samples represents the transcriptome of PAC. RNA yields from LCM-derived samples are commonly very low. To obtain a working concentration, extracted RNA was amplified and three replicates per tissue type were analyzed.

The number of reads contained in each library was greater than 2.9 × 10^7^ and only 25 to 46.9 % reads of the reads were mapped to the genome uniquely, where both pairs of reads mapped in the expected direction and with the expected distance between them (concordantly). Most of the other reads were mapped to multiple locations in the genome. Of the approximately 39 k genes contained in the potato genome, 15 to 23 k genes were detected in the three replicates of phloem-associated cells (either petiole-PAC or stem-PAC) (Additional file 1: Table S1). Most of the genes that were expressed in only one or two of the replicates are present at very low abundance (<10 reads, for example). The six libraries were normalized with the upper quantile and analyzed with a Generalized Linear Model using QuasiSeq. *P*-values were calculated with QLSpline based on negative binomial distribution. *Q*-values were given by adjusting the *p*-value for familywise error rate (Additional file 2: Figure S1). After removing the effect of the sequencing method, a mean value was calculated for petiole-PAC and stem-PAC to indicate their measured level. All of the genes identified in the transcriptomes of this report with mean read values are listed in Additional file 3: Table S2. Excluding the low read hits (<10 reads), 15,167 genes can be identified in either petiole-PAC or stem-PAC transcriptome (Group 2 under the whole genome column, Table 1). The number of expressed genes are comparable to the 14,242 and 13,775 genes identified in the vascular bundles of cucumber and watermelon, respectively [36].

**Table 1:**
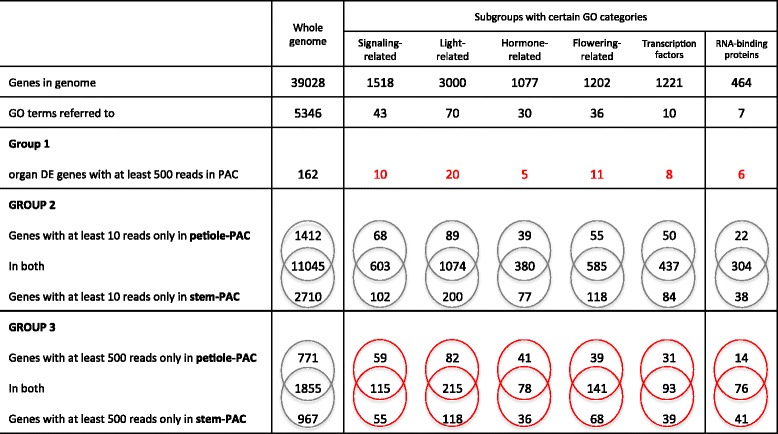
Distribution of gene expression in different gene ontology categories for both differentially expressed (Group 1) and unique genes of petiole- and stem-PACs

GO terms were searched from the AmiGO 2 GO browser. GO analysis of the potato genome was performed with Blast2GO. Light-related and hormone-related were excluded from the signaling-related category

### Comparison of Petiole-PAC and stem-PAC transcriptomes

Out of the 26,898 genes that exhibited any expression in either petiole-PAC or stem-PAC, 2087 were identified as differentially expressed (DE) genes between petiole-phloem and stem-phloem, with *q*-values less than 0.05 (Additional file 4: Table S3). Most of these genes are expressed at low abundance levels (<10 mean value in both petiole-PAC and stem-PAC). Only 573 DE genes have a mean value >10 in either petiole-PAC or stem-PAC (Additional file 4: Table S3). At the 500 read cutoff level, the number of DE genes between petiole-PAC and stem-PAC is only 162 (Table 1, Group 1). This suggests that the petiole PAC and stem PAC have very similar transcriptomes.

**Fig. 2:**
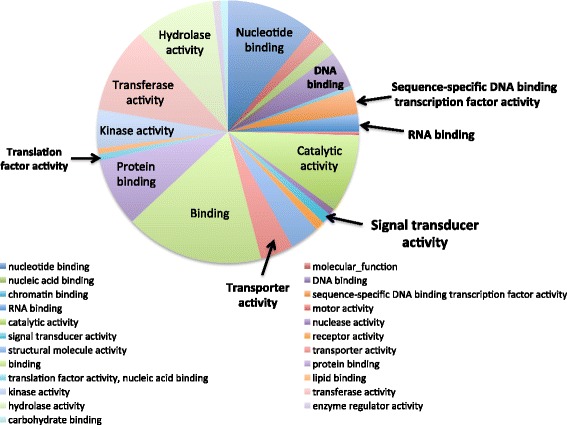
Distribution of molecular functions in the differentially expressed genes between petiole-PAC and stem-PAC. Differentially expressed (DE) genes were identified with generalized linear model by using QuasiSeq [93]. Genes with less than ten reads in both petiole-PAC and stem-PAC were removed because of their low abundance. GO terms of the whole genome were analyzed with Blast2GO. The GO categories identified for each gene were made comparable by converting each GO term to the same level in the GO structure to permit. This was done with goslimviewer in AgBase [96] (http://agbase.msstate.edu/cgi-bin/tools/goslimviewer_select.pl)

To visualize functional relationships in these expression profiles, an ontological analysis was performed. Gene ontology (GO) categories of all the genes in potato were obtained from the GO database using Blast2GO, with parameters of 20 hits and an e-value of 10e^−6^. 22,058 genes out of the 39,028 genes (56.5 %) in the potato genome were matched with at least one GO term. The 573 DE genes were mapped to 3791 GO terms including 736 molecular functions, 2579 biological processes, and 476 cellular components (Fig. 2). As we would expect with active transport and loading through the phloem cells, binding function is one of the most prominent functions in the genes expressed in PAC. Out of the 736 molecular functions, 81, 62, and 126 were classified as “nucleotide binding”, “protein binding”, and “binding”, respectively (Fig. 2). “DNA binding” and “RNA binding” have lower numbers, 33 and 16 respectively, but these functions have very important roles in transcription, mRNA stabilization, localization and transport (Fig. 2, arrows). The signaling-related biological processes and molecular functions were more closely examined for all unique and DE genes related to signal transport, a prominent function of phloem (Table 1). Specifically, the transcripts encoding proteins functional in signaling and regulation, such as light-induced signaling, photoperiodism, floral induction, hormone-related signaling and transcription factors, were explored due to their importance in long-distance transport. Among these DE “signal” genes abundantly expressed, 10 are classified as signaling-related, 20 as light-related, 5 as hormone-related, and 11 as flowering-related (Group 1, Table 1). Eight genes are classified as transcription factors and six encode for RNA-binding proteins. DE genes from signaling, light, hormone, flowering, and RNA-binding GO categories are listed in Table 2.

**Table 2:** Differentially expressed genes between petiole-PAC vs. stem-PAC with select gene ontology categories

Related function	Signaling-related genes	Petiole phloem	Stem phloem	Long day mean	Short day mean	Organ effect. *Q*-value	Photoperiod *Q*-value	Annotation
Signaling	PGSC0003DMG400000584	1211	366	486	1487	1.99E-02	6.80E-10	Pseudo-response_regulator_5
Signaling	PGSC0003DMG400000792	1337	335	775	710	3.12E-02	7.75E-02	Ran_GTPase_binding_protein
Signaling	PGSC0003DMG400001272	449	1474	15,250	16,791	4.78E-02	1.69E-02	ADP-ribosylation_factor_1
Signaling	PGSC0003DMG400002613	1949	941	3162	3697	2.42E-02	7.82E-02	PDR8_PEN3_(PLEIOTROPIC_DRUG_RESISTANCES)
Signaling	PGSC0003DMG400005792	115	504	114	112	1.57E-02	3.37E-01	Nucleoside_diphosphate_kinase
Signaling	PGSC0003DMG400016822	761	55	360	340	1.30E-02	1.45E-01	ADP-ribosylation_factor
Signaling	PGSC0003DMG400017350	62	604	454	452	3.29E-02	3.65E-01	ATP binding_protein
Signaling	PGSC0003DMG400021253	1936	691	2978	2746	3.21E-02	6.53E-02	Mitogen-activated_protein_kinase
Signaling	PGSC0003DMG400023211	188	538	2670	4268	2.75E-02	2.65E-07	Phospholipase_C
Signaling	PGSC0003DMG400028694	1132	330	1330	975	4.05E-02	1.67E-05	Ethylene receptor homolog
Light	PGSC0003DMG400000584	1211	366	486	1487	1.99E-02	6.80E-10	Pseudo-response_regulator_5
Light	PGSC0003DMG400000792	1337	335	775	710	3.12E-02	7.75E-02	Ran_GTPase_binding_protein
Light	PGSC0003DMG400001299	41	883	1418	1372	3.29E-02	2.11E-01	Ankyrin_repeat_domain_protein
Light	PGSC0003DMG400001342	894	149	1299	1288	3.44E-04	3.60E-01	Conserved_gene_of_unknown_function
Light	PGSC0003DMG400005792	115	504	114	112	1.57E-02	3.37E-01	Nucleoside_diphosphate_kinase
Light	PGSC0003DMG400007966	237	630	1991	2222	1.15E-02	1.11E-02	Annexin
Light	PGSC0003DMG400008589	705	354	2624	2510	2.15E-02	9.22E-02	RNA_helicase
Light	PGSC0003DMG400010794	322	1756	3137	2419	3.40E-02	6.32E-05	Cellulose_synthase
Light	PGSC0003DMG400018104	2304	870	2852	1442	4.36E-02	6.46E-10	Cell_division_cycle_protein_48
Light	PGSC0003DMG400018449	5071	8343	15,036	18,618	3.26E-02	2.70E-05	Actin
Light	PGSC0003DMG400018795	1366	33	79	100	1.22E-02	3.01E-02	Multidrug_resistance_protein_1,_2
Light	PGSC0003DMG400020086	1604	601	3090	3130	3.00E-02	3.16E-01	26S_proteasome_subunit_4
Light	PGSC0003DMG400022381	1357	650	2758	3376	2.39E-03	1.07E-04	Conserved_gene_of_unknown_function
Light	PGSC0003DMG400024249	782	1654	459	835	7.52E-03	1.02E-08	Gibberellin_20-oxidase-l
Light	PGSC0003DMG400026500	322	792	17,945	19,242	4.43 E-02	5.04E-02	Type_1_(26_kD)_CP29_polypeptide
Light	PGSC0003DMG400029829	2102	3550	9228	8869	1.07E-02	9.70E-02	Eukaryotic_initiation_factor_3E_subunit
Light	PGSC0003DMG400030867	1073	2924	6003	5469	1.67E-02	5.99E-02	Acyl-CoA-binding_protein
Light	PGSC0003DMG400031124	611	1050	2412	2959	4.85E-02	2.95E-02	Myosin_Xl-F
Light	PGSC0003DMG400031812	60	515	1556	1554	2.89E-02	3.70E-01	DNA_photolyase
Light	PGSC0003DMG400035320	18	668	222	221	1.92E-03	3.70E-01	F-box_leucine_rich_repeat_protein
Hormone	PGSC0003DMG400001342	894	149	1299	1288	3.44E-04	3.60E-01	Conserved_gene_of_unknown_function
Hormone	PGSC0003DMG400009773	540	1364	2414	2546	1.50E-02	2.34E-01	Auxin_response_factor_19
Hormone	PGSC0003DMG400010794	322	1756	3137	2419	3.40E-02	6.32E-05	Cellulose_synthase
Hormone	PGSC0003DMG400014452	966	346	1795	1904	3.34E-02	1.33E-01	Auxin_response_factor 2
Hormone	PGSC0003DMG400028694	1132	330	1330	975	4.05E-02	1.67E-05	Ethylene_receptor_homolog
Flowering	PGSC0003DMG400001453	3296	798	1306	1529	2.91E-02	5.66E-04	RNA-binding_protein
Flowering	PGSC0003DMG400029829	2102	3550	9228	8869	1.07E-02	9.70E-02	Eukaryotic_initiation_factor_3E_subunit
Flowering	PGSC0003DMG400018795	1366	33	79	100	1.22E-02	3.01E-02	Multidrug_resistance_protein_1,_2
Flowering	PGSC0003DMG400014452	966	346	1795	1904	3.34E-02	1.33E-01	Auxin_response_factor_2
Flowering	PGSC0003DMG400024249	782	1654	459	835	7.52E-03	1.02E-08	Gibberellin_20-oxidase-l
Flowering	PGSC0003DMG400032166	567	115	669	718	2.32E-02	5.94E-02	Lysyl-tRNA_synthetase
Flowering	PGSC0003DMG400017035	500	1292	2559	2434	1.76E-02	2.74E-01	RAPTOR1B
Flowering	PGSC0003DMG400002895	44	1252	196	222	2.09E-02	4.14E-02	Sucrose_synthase
Flowering	PGSC0003DMG400004634	222	1178	419	480	3.17E-02	1.40E-02	Sentrin_sumo-specific_protease
Flowering	PGSC0003DMG400008366	206	1140	545	673	3.84E-02	3.83E-03	FI ACA_ribonucleoprotein_complex_subunit
Flowering	PGSC0003DMG400008431	211	641	573	612	7.02E-03	1.00E-01	Protein_arginine_n-methyltransferase_1
RBPs	PGSC0003DMG400007507	1166	443	1297	1422	4.23E-02	1.47E-02	3-5-exoribonuclease_RNA_binding
RBPs	PGSC0003DMG400021249	110	508	1413	1388	6.95E-03	2.93E-01	RNA-binding_protein
RBPs	PGSC0003DMG400022220	293	1083	7037	7840	4.86E-02	2.45E-03	RNA_Binding_Protein_45
RBPs	PGSC0003DMG400023660	79	567	835	814	4.17E-02	2.29E-01	RBP50

### Unique and DE genes of petiole-PAC and stem-PAC

Even though petiole- and stem-PAC transcriptomes are similar, there are several transcripts that were expressed uniquely in each. The differentially and uniquely expressed genes indicate the slight difference between petiole phloem and stem phloem. With 10 reads as a mean threshold, approximately 11 k of the genes are expressed in both petiole-PAC and stem-PAC (Table 1, whole genome column, Group 2). There are 1412 and 2710 unique genes expressed in petiole-PAC and stem-PAC, respectively (Table 1, Group 2). Few of these are highly expressed exemplified by the fact that there are only ten petiole-PAC unique genes with greater than 500 reads (Table 3), and only 26 in stem-PAC (Table 4). Several of these highly expressed PAC genes have been functionally characterized in other organisms, such as AP2, an ERF domain-containing transcription factor [37, 38], sucrose synthase [39, 40], and several other DNA- or RNA-binding proteins. The pentatricopeptide repeat-containing protein (PPR) is a RNA-binding protein essential for RNA editing in chloroplasts and mitochondria [41, 42]. PPR proteins help to restore fertility to cytoplasmic male-sterile plants [43] and are involved in organelle biogenesis [44]. Also included in the signaling category, are FRIGIDA, a scaffolding protein involved in flowering, that functions in the formation of a complex that includes both general transcription and chromatin-modifying factors [45] and a jmjC-domain protein. A rice jmjC-domain protein functions in controlling suppression of flowering [46].

**Table 3:** Genes uniquely expressed in petiole phloem-associated cells

	Petiole phloem	Stem phloem	P-value	Q-value	Annotation
PGSC0003DMG400008734	661	0	0.000	0.001	AP2_ERF_domain-containing_transcription_factor
PGSC0003DMG400010442	576	1	0.003	0.017	Pentatricopeptide_repeat-containing_protein
PGSC0003DMG400018147	760	5	0.072	0.179	Gene_of_unknown_function
PGSC0003DMG400025480	593	7	0.059	0.163	Receptor_protein_kinase
PGSC0003DMG400030897	674	3	0.016	0.075	Gene_of_unknown_function
PGSC0003DMG400036011	671	4	0.026	0.103	Gene_of_unknown_function
PGSC0003DMG401005729	531	7	0.316	0.331	Cell_wall-associated_kinase
PGSC0003DMG401011335	733	7	0.268	0.311	UDP-glucuronic_acid_decarboxylase_2
PGSC0003DMG401025754	886	9	0.088	0.196	Gene_of_unknown_function
PGSC0003DMG402003286	524	7	0.005	0.033	ACI112

**Table 4:** Genes uniquely expressed in stem phloem-associated cells

	Petiole phloem	Stem phloem	P-value	Q-value	Annotation
PGSC0003DMG400001320	2	769	0.006	0.036	Alpha-tubulin
PGSC0003DMG400001999	2	621	0.097	0.206	BZIP_protein
PGSC0003DMG400002009	8	746	0.005	0.032	Hsp20_alpha_crystallin_family_protein
PGSC0003DMG400002303	7	1631	0.003	0.019	Glucan_endo-l,3-beta-glucosidase
PGSC0003DMG400002728	1	581	0.155	0.251	Homeodomain
PGSC0003DMG400006943	6	712	0.242	0.299	Cation_efflux_family_protein
PGSC0003DMG400009942	1	561	0.144	0.245	Gene_of_unknown_function
PGSC0003DMG400011331	6	536	0.118	0.225	Dynein_light_chain
PGSC0003DMG400013186	5	985	0.263	0.309	Ubiquitin_carboxyl-terminal_hydrolase
PGSC0003DMG400015598	2	681	0.059	0.162	Conserved_gene_of_unknown_function
PGSC0003DMG400019310	4	826	0.263	0.309	Jumonji (jmjC)-domain containing_protein
PGSC0003DMG400019353	2	564	0.108	0.217	Conserved_gene_of_unknown_function
PGSC0003DMG400020660	4	660	0.101	0.211	Protein_kinase_domain_containing_protein
PGSC0003DMG400023407	3	715	0.233	0.296	RRM-containing_protein
PGSC0003DMG400026029	1	508	0.006	0.034	MetalloendopepVdase
PGSC0003DMG400026879	3	821	0.080	0.188	Ubiquitin-associated_TS-N_domain-containing protein
PGSC0003DMG400028078	9	547	0.030	0.113	Metalloendopeptidase
PGSC0003DMG400029153	7	807	0.045	0.141	Amino_acid_transporter
PGSC0003DMG400030178	5	817	0.122	0.228	Gene_of_unknown_function
PGSC0003DMG400030396	1	574	0.026	0.105	Conserved_gene_of_unknown_function
PGSC0003DMG400030555	3	537	0.143	0.244	Acetylglucosaminyltransferase
PGSC0003DMG400031046	6	3416	0.006	0.033	Sucrose_synthase
PGSC0003DMG400033575	5	1292	0.186	0.272	Poly(RC)-binding_protein
PGSC0003DMG400034493	4	588	0.043	0.138	FRIGIDA
PGSC0003DMG401023562	1	647	0.108	0.216	Ubiquitin_thiolesterase
PGSC0003DMG403024767	2	557	0.004	0.027	Pectinesterase

Gene ontology categories analyzed for the DE genes were also considered for the uniquely expressed genes in petiole-PAC and stem-PAC (Table 1, Group 2). With 10 reads as a cut-off, the genes uniquely expressed in the stem-PAC are approximately two-fold more in number than the genes uniquely expressed in the petiole-PAC in each category (Table 1, Group 2). Whereas, when considering only the most abundant RNAs (>500 average reads), the number of uniquely expressed genes from both sources is comparable (Table 1, Group 3). These latter abundant transcripts are plausibly important regulators of phloem function or mobile transcripts present in the sieve elements. In summary, there are approximately 1000 genes in these groups (signaling, light, hormone, flowering, transcription factors, RNA-binding) including both unique and common (Table 1, Group 3, red ovals). A complete list of these genes can be found in Additional file 5: Table S4.

Among these genes in the select GO categories of Table 1, many are important regulators of development that may provide insights into the differences between petiole-PAC and stem-PAC. Pseudo-response regulator 5 (PGSC0003DMG400000584) is a well-characterized gene, which has an important role in circadian rhythm [47]. It is classified as a DE petiole-PAC gene, with 1211 reads in the petiole-PAC and only 366 reads in the stem-PAC. BEL33 (PGSC0003DMG400024267) is a flowering-related gene in the TALE (Three Amino Acid Loop Extension) superfamily [48]. Its expression level in stem-PAC is almost twice that of petiole-PAC levels. Gibberellin_receptor GID1 (PGSC0003DMG400028559) is a GA receptor that regulates hormone responses [49]. It is expressed in petiole-PAC with a mean of 1017 reads, five times more than levels in stem-PAC. BRI1 protein is a leucine-rich repeat protein localized to the membrane with an extracellular brassinosteroid receptor domain and intracellular kinase domain [50] and functions in controlling the autonomous flowering pathway [51]. The ccr4-associated protein has mRNA deadenylation activity and is functional in defense [52]. The chromo-domain protein, LHP1, is involved in epigenetic silencing of target genes such as flowering genes. Genetic experiments have shown that LHP1 can affect flowering time and vegetative growth [53].

RNA-binding proteins (RBP) interact with transcripts to mediate numerous aspects of RNA metabolism [54] and function as chaperones that facilitate the long-distance transport of phloem-mobile mRNAs [20]. In the transcriptome of PAC, 364 out of the 464 RBPs in the potato genome were detected (Additional file 6: Table S5). Several of these RBPs have been documented in the literature. The pumpkin ortholog of StPTB1 and StPTB6 (Table 5) was identified as the core protein in a mobile nucleoprotein complex in pumpkin phloem [20]. StPTB1 and StPTB6 play important roles in regulating the movement of the mobile RNA, *StBEL5* in potato [55]. IF2 is the potato ortholog of Nova, a KH domain RBP, that binds to StPTB1 and StPTB6 [56]. A PTB7-like protein was also identified in pumpkin phloem and its orthologs in *Arabidopsis* have been implicated in alternative splicing [2, 57]. Several of the RBPs identified using the 3′ UTR of *StBEL5* as bait in the yeast three-hybrid system [58] were also detected in the petiole- and stem-PAC transcriptomes (Table 5). These include sucrose synthase, eIF5A, and a glycine–rich RBP. Four pumilio proteins containing a Puf domain that interacts with 3′ UTRs in target RNAs were detected. All four were relatively abundant in both petiole and stem profiles. Pumilio has only recently been discovered in plants but is widespread in numerous species and functions in diverse aspects of mRNA metabolism that regulate development and defend against viruses [59–61].

**Table 5:** Abundance of RNA-binding proteins in phloem-associated cells

Gene	Gene ID	Petiole phloem	Stem phloem	Identified in	Citation
StPTBl	PGSC0003DMG400018824	234	373	Ortholog of CmRBP50	Ham et al., [20]; Butler et al. [35]
StPTB6	PGSC0003DMG400023660	79	567	Ortholog of CmRBP50	Ham et al., [20]; Butler et al., [35]
StPTB7	PGSC0003DMG400001427	140	188	In potato PTB family	Rühl et al., [57]
StPTB7-l	PGSC0003DMG400009106	28	43	In potato PTB family	Rühl et al., [57]
StPTB7-2	PGSC0003DMG400002353	18	16	In potato PTB family	Rühl et al., [57]
StPTB7-3	PGSC0003DMG400019613	101	391	In potato PTB family	Rühl et al., [57]
RBP1	PGSC0003DMG402017409	652	1005	pumpkin phloem proteome	Lin et al., [2]
GRP7	PGSC0003DMG400000708	99,744	161,520	pumpkin phloem proteome	Lin et al., [2]
RBP-45	PGSC0003DMG400011290	1217	724	pumpkin phloem proteome	Lin et al., [2]
RBP6	PGSC0003DMG400012601	2747	2147	pumpkin phloem proteome	Lin et al., [2]
Albal	PGSC0003DMG400020480	1	64	pumpkin phloem proteome	Lin et al., [2]
Alba2	PGSC0003DMG400020460	0	43	pumpkin phloem proteome	Lin et al., [2]
Pumiliol	PGSC0003DMG400002143	4748	5411	Leaf development	Huang et al., [61]
Pumilio2	PGSC0003DMG400006350	344	669	Potato PAC	Abbasi et al., [59]
Pumilio3	PGSC0003D MG400009166	834	343	Potato PAC	Abbasi et al., [59]
Pumilio4	PGSC0003DMG400030327	751	1109	Potato PAC	Abbasi et al., [59]
IF2 (Nova)	PGSC0003DMG400023482	227	329	pumpkin phloem Y2H with PTB1 and PTB6	Lin et al., [2]; Shah et al., [56]
IF1 (RRM protein)	PGSC0003DMG400008877	600	1353	Y2H with PTB1 and PTB6	Shah et al., [56]
Sucrose svnthase-4	PGSC0003DMG400013546	4219	7886	Y3H with StBEL5 3′ UTR	Cho et al., [58]
GR-RBP3	PGSC0003DMG400028111	345	355	Y3H with StBEL5 3′ UTR	Cho et al., [58]
LSH10	PGSC0003DMG400020442	44	22	Y3H with StBEL5 3′ UTR	Cho et al., [58]
elF5A	PGSC0003DMG400011137	1077	1216	Y3H with StBEL5 3′ UTR	Cho et al., [58]
B5RBP5	PGSC0003DMG400031406	352	527	Y3H with StBEL5 3′ UTR	Cho et al., [58]
Zinc finger	PGSC0003D MG400027176	4424	1879	Y3H with StBEL5 3′ UTR	Cho et al., [58]
B5RBP7	PGSC0003DMG400017156	4323	5245	Y3H with StBEL5 3′ UTR	Cho et al., [58]

**Fig. 3:**
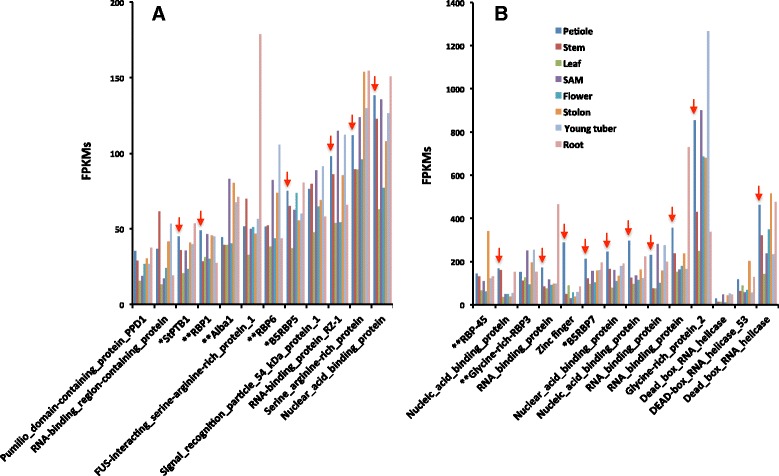
Expression profiles of select RNA-binding proteins mined from RNA-seq data from the Potato Genome Database from the Tuberosum RH89-039-16 haplotype [62]. RNA profiles from eight organs are presented and medium (**a**) and high (**b**) abundance values are shown in FPKMs (fragments per kb per million mapped reads). Selection of these RNAs was based on their relative abundance in petiole-PACs, their binding affinity for the 3′ UTR of the mobile RNA, StBEL5* [58], or the presence of their protein orthologs in pumpkin phloem sap** [2]. Red arrows designate petiole samples. Accession numbers for these genes are listed in Additional file 7: Table S6

To validate expression patterns of select RBPs without the bias of amplification or the imbedding techniques inherent in the LCM protocol, a sample of RNA profiles from the Potato Genome Sequencing Consortium [62] was assembled in eight different organs for 26 RBPs (Fig. 3). These were selected on the basis of their relative abundance in petiole-PACs, their binding affinity for the 3′ UTR of the mobile RNA, *StBEL5* [58], or the presence of their protein orthologs in pumpkin phloem sap [2]. Consistent with their abundance in petiole-PAC, most of these proteins exhibited significant levels of transcripts in petioles (Fig. 3, red arrows). Among the samples, three RNA helicases, one PTB protein, and two glycine-rich RBPs were profiled. Plant helicases have a known function in regulating the size exclusion limit of plasmodesmata [63]. Several RBPs exhibited spikes in transcript levels in specific organs (Fig. 3; Additional file 7: Table S6). RBP6, RBP RZ-1, and glycine-rich protein 2 were relatively abundant in young tubers. RBP-45 and one of the Dead-box RNA helicases exhibited select relative abundance of transcripts in stolons. Two of the “RNA-binding proteins” and the FUS-interacting ser/arg-rich protein-1 all spiked in roots. These observed abundance levels may reflect a putative organ-specific function for select RBPs.

### Abundant transcripts in phloem-associated cells

Transcriptome profiling identified a plethora of genes that are highly expressed in petiole PAC or stem PAC. The highest average total reads for one specific gene were 154,207 in petiole PAC and 274,582 in stem PAC. In the petiole-PAC library, 1209 genes had >1000 reads and 2626 genes had >500. In the stem-PAC library, 1288 genes had >1000 reads and 2822 genes had >500. There are 3593 genes with >500 reads in either petiole-phloem or stem-phloem associated cells (Additional file 8: Table S7). To compare the genes highly expressed in PAC with other genes in the whole genome, these 3593 genes were regarded as genes with abundant transcripts and were analyzed for attributes based on gene annotations.

**Fig. 4:**
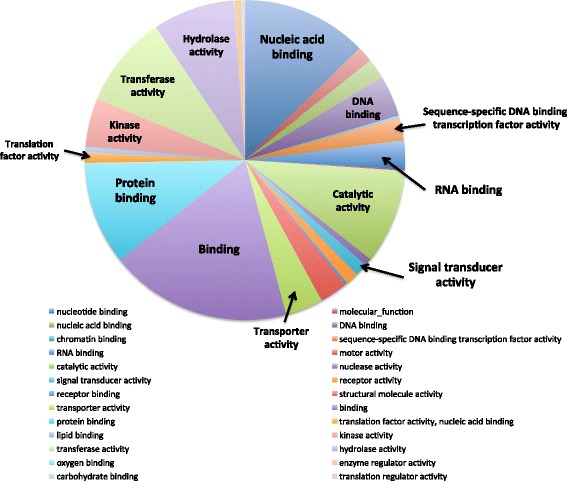
Distribution of molecular functions in the abundant transcripts in phloem associated cells. The reads of all the genes identified in petiole-PAC or stem-PAC were normalized with upper quantile normalization. A mean value is calculated from the three replicates. Genes with more than 500 reads in the mean value of either petiole-PAC and stem-PAC were regarded as abundantly expressed genes. GO terms of the whole genome were analyzed with Blast2GO. The GO categories identified for each gene were made comparable by converting each GO term to the same level in the hierarchy to permit clustering into GOslim categories. This was done with goslimviewer using AgBase software [96] (http://agbase.msstate.edu/cgi-bin/tools/goslimviewer_select.pl)

**Fig. 5:**
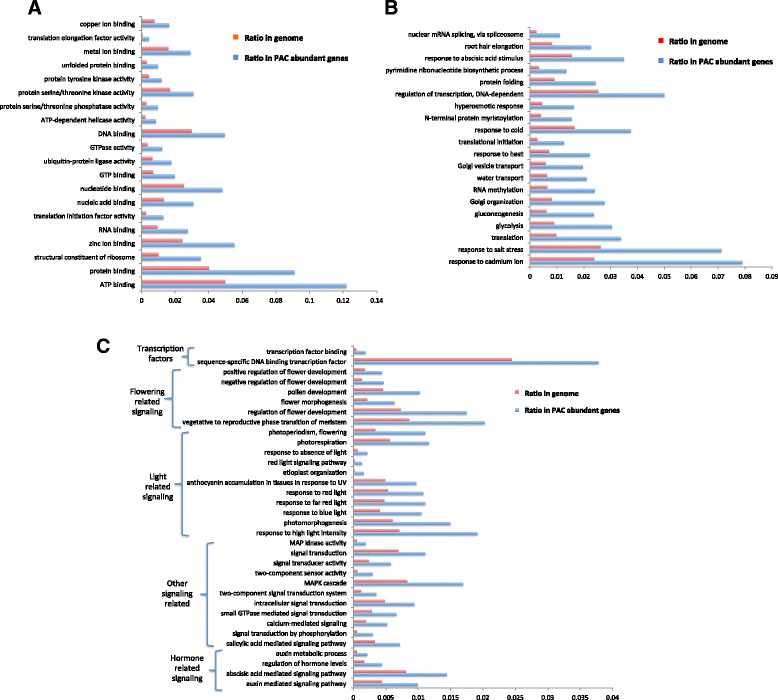
Over-represented molecular function and biological processes in phloem abundant transcripts. GO terms involved with DE genes between petiole-PAC and stem-PAC were analyzed with GOseq [64] to compare their enrichment in DE genes relative to the whole genome. The *p*-value is adjusted with BH method [97]. The over-represented genes were defined with adjusted *p*-value smaller than 0.05. The ratio of each GO term is calculated by dividing the number of genes involved with each GO term with number of genes in the whole group. **a** includes the top 20 over-represented GO terms of molecular function and (**b**) includes the top 20 over-represented GO terms of biological processes. **c** shows the GO terms related to transcription factor, flowering, light, signaling and hormone that are over-represented

Approximately 2971 GO terms were involved with the abundant transcripts in PAC. The GO categories identified for each gene were made comparable by converting each GO term to the same level in the hierarchy to permit clustering into GOslim categories. This was done with goslimviewer using AgBase software. Twenty-five cellular components, 44 biological processes and 26 molecular functions were applied (Fig. 4). The most abundant GO categories represented were “binding” and “nucleic acid binding” (Fig. 4). By comparing to the whole genome with GOseq [64], the gene ontology of the abundant transcripts revealed 511 categories over-represented with adjusted *p*-values smaller than 0.05. In the 510 over-represented GO categories there are 101 molecular functions, 73 cellular components and 336 biological processes. Many binding-related functions were verified as over-represented categories with adjusted *p*-values smaller than 0.05. Ion binding functions, such as zinc ion binding, copper ion binding, cobalt ion binding and calcium ion binding, were all over-represented (Fig. 5a). RNA-binding and protein-binding functions were also over-represented in active PAC genes. These binding functions likely contribute to facilitating the transport processes of phloem. The PAC-abundant transcripts are also involved with numerous important biological processes, including both response and developmental activities (Fig. 5b-c). The top 32 over-represented molecular function and biological processes related to signaling included responses to both light quality and quantity (Fig. 5c). These signals are commonly perceived in the leaf, and phloem in the leaf veins and petiole serve as the conduit to deliver these signals to other organs. There are also several flowering-related GO categories over-represented in the abundant transcripts, including photoperiodism, flowering, ovule development, regulation of flower development, and flower morphogenesis (Fig. 5c). Among the 175 GO categories related to signaling, as listed in Table 1, 34 of them are over-represented in the PAC-abundant transcripts (all listed in Fig. 5c).

### The effect of photoperiod on the transcriptome of petioles

Day length regulates numerous aspects of plant development and is especially important in potato for controlling tuber formation. The perception of length of daylight/darkness generates leaf-derived signals that are traansported throughout the whole plant through petiole/stem vascular connections [9, 13, 14]. To identify genes that are regulated in petioles in response to photoperiod, we sequenced RNA samples from petioles of the photoperiod-responsive species *Solanum tuberosum* ssp. *andigena* under long-day (LD) and SD photoperiods using RNA-seq. Four replicate samples for each were isolated from the petiole tissues harvested from plants grown under long- and short-day conditions. The reads were mapped to the potato genome with GSNAP, and the number of concordant unique reads was counted for each gene using HT-seq (Additional file 9: Table S8). The number of reads contained in each library was greater than 1 × 10^7^ and approximately 94 % of the paired reads were mapped to the genome in a concordant and unique manner. Of the 39,028 genes contained in the potato, approximately 25 k genes were detected in the whole petiole samples (LD Petiole and SD Petiole) with at least one read aligning to the gene (Additional file 9: Table S8). Representing only a few cell types from the petiole and stem organs, RNA-seq results of PAC scored significantly fewer genes than the whole petiole sample (Additional file 1: Table S1). The genes expressed in phloem are very likely detected in the whole petiole samples depending on the depth of the petiole profile. Beyond PAC, there are many other cell types in the petiole (e.g., collenchyma, sclerenchyma, palisade parenchyma, and epidermis), so a proportion of genes will likely only be detected in the whole petiole. In theory, the genes with higher reads in PAC represent genes with a significant putative function in the phloem. Of course, this set of genes will also be detected in the whole petiole samples, albeit at a lower proportion of reads (Additional file 3: Table S2).

**Fig. 6:**
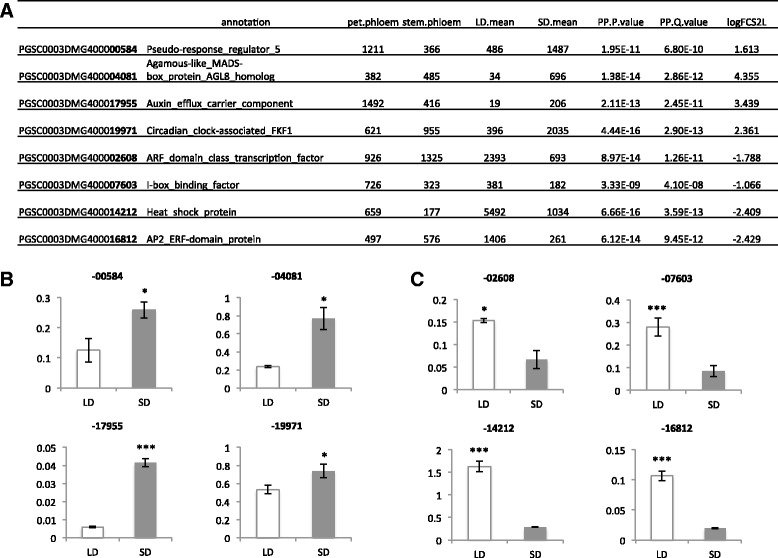
Select genes that are differentially expressed in petioles of potato in response to photoperiod (**a**). Mean reads from RNA-seq data are presented for both petiole- and stem-PAC and for LD/SD petioles. Real time RT-PCR was used to verify the RNA-seq results for SD-induced (**b**) or LD-induced (**c**) genes. Each gene in panels **b** and **c** is designated by its 5-digit PGSC identifier (in *bold*, **a**). Relative levels of their transcripts were quantified using total RNA extracted from petiole samples harvested from plants grown under long (*open bars*) or short (*shaded bars*) days. Short-day plants were harvested after 10day of SD conditions (8 h light, 16 h dark). Quantitative real-time RT-PCR with gene-specific primers was used to calculate the relative amounts of RNA for each gene. The expression of each gene was calculated as the 2^-ΔCt^ value and normalized to the endogenous reference gene, *StAct8*. Standard errors of the means of three biological replicates are shown with *1* and *3 asterisks* indicating significant differences (*p* < 0.05, *p* < 0.001, respectively) using a Student’s *t* test

Reads of the eight libraries were normalized using the upper quantile normalization approach. A generalized linear model was applied to the LD Petiole and SD Petiole results with the R package “QuasiSeq” to analyze the photoperiod effect. All other conditions were kept consistent and the libraries were sequenced with multiplexing tag in the same lane, so photoperiod effect is the only fixed effect that was considered (Additional file 10: Figure S2). Eleven-thousand, nine-hundred, fifty-seven genes were identified as significantly DE with *q*-values less than 0.05 (Additional file 11: Table S9). Means of the normalized reads of the four replicate libraries for both long- and short-day treatments were used to indicate their measured level. Among the 20,564 genes with at least 10 reads in either LD or SD petiole, 517 of them are uniquely expressed in LD, and only 388 of them are uniquely expressed in SD. Most of these uniquely expressed genes exhibited low abundance read values. The most abundant transcript uniquely expressed in LD has only 558 reads (PGSC0003DMG400026590) whereas the most abundant unique transcript under SD conditions has only 208 reads (PGSC0003DMG400016462). Among the 11,957 DE genes, 5555 of them are up-regulated under LD, and 6402 of them under SD. Twelve-hundred and eight of the DE genes activated by LD have a log2-fold change more than 1, and 248 genes out of the 1208 have reads more than 500 under LD (Additional file 12: Table S10). Eight-hundred and twenty of the DE genes activated by SD have a log2-fold change more than 1, and 128 genes out of the 820 have reads more than 500 under SD (Additional file 13: Table S11). Transcripts that are regulated by photoperiod and are relatively abundant (>380 reads) in petiole-PAC may be indicative of genes involved in signaling or transport mediated by day length. Included in this list are genes encoding for the Agamous-like MADS-box protein/AGL8 ortholog, a circadian clock-associated FKF1 protein, Pseudo-response regulator 5, the AP2 ERF-domain protein, an ethylene receptor, a NAC-domain protein, and a nodulin MtN3 family protein (Additional file 12: Table S10 and Additional file 13: Table S11). As an example, the AGL8 ortholog of potato is induced by the StFT-like tuberization signal, SP6A [14], and is involved in controlling meristem and tuber development by regulating cytokinin levels [65]. Several notable DE photoperiod genes were selected to verify their relative expression levels with qRT-PCR (Fig. 6a). Four were up-regulated by SD (Fig. 6b) and four by LD (Fig. 6c). All eight exhibited expression patterns consistent with their comparable RNA-seq data.

### Gene ontology of photoperiod-regulated genes of the petiole

**Fig. 7:**
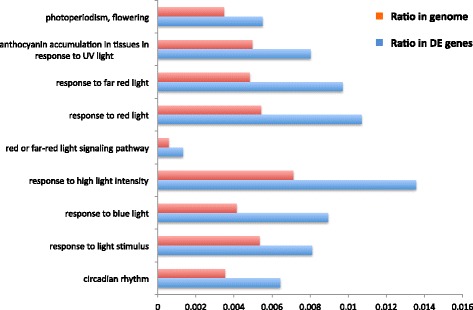
Over-represented GO terms related to light signaling in DE photoperiod genes. GO terms involved with DE genes between long- and short-day treatments were analyzed with GOseq [64] to compare their enrichment in DE genes relative to the whole genome. The *p*-value was adjusted with BH method [97]. The over-represented genes were defined with adjusted *p*-value smaller than 0.05. The ratio of each GO term is calculated by comparing the number of genes involved with each GO term with number of genes in the whole group

To visualize functional relationships in this diverse expression profile, the 11,957 DE genes were also analyzed for their gene ontology categories. Four-thousand, four-hundred, and twenty-nine GO terms were applied to the 11,957 photoperiod DE genes, including 6056 of cellular component, 34,632 of biological process and 29,235 of molecular function. GO distribution of the photoperiod DE genes was analyzed with GOseq to identify the over-represented GO groups. With familywise adjusted *p*-value <0.05, GO terms for 64 cellular components, 136 molecular functions, and 369 biological processes were significantly enriched in the photoperiod DE genes (Fig. 7; Additional file 14: Figure S3 and Additional file 15: Figure S4). As expected, “circadian rhythm” is identified as an over-represented GO category as well as several light-related GO terms (Fig. 7). Among the molecular functions, binding is the most over-represented function, including binding to ATP, protein, nucleotide, DNA, RNA, and several kinds of ions (Additional file 14: Figure S3).

### RNA-binding motifs

Mobility of mRNA, stability and control of translation are facilitated by RNA-binding proteins associated with them. RBPs commonly bind to conserved elements in the 3′ un-translated region (UTR) of the RNA. To assess the frequency of select RBP motifs in potato transcripts, downstream sequence (DSS) from the stop codon was screened for the presence of RBP motifs. As a reference, in *Arabidopsis*, the average length of the 3′ UTR is 248 nt [66]. Three known RBP target elements were searched, including those for polypyrimidine tract-binding proteins (PTB), Pumilio and Nova, a KH-domain protein [67] (Additional file 16: Figure S5A-C). Pumilio was selected because of its relative abundance in PAC (Table 5), its functional relevance, and its widespread role in RNA metabolism [68]. PTB and Nova were selected because of their prominence in binding to RNAs and because both were detected in phloem sap of cucumber suggesting they are both phloem mobile [2]. The Pumilio binding motif has been confirmed as UGUAu/c/aAUA where the 5th nucleotide can be U, C, or A [69], whereas Nova’s is modeled as u/c/aCAUUUCAc/u [67]. PTB proteins bind to RNA at four RNA recognition motifs (RRM). Each RRM can interact with a cytosine/uracil (CU) motif ranging from 3 to 6 nt. In our search, the PTB motif was defined as a cluster of four CU runs each, at least, 4 nt in length within the designated DSS. Biochemical analysis of interactions of target RNA to the binding pockets of PTB protein domains demonstrated that binding to PTBs is not sequence-specific and that many RNA fragments readily bind to them [70].

Using the MEME suite [71] and BEDTools [72], Pumilio, Nova, and PTB binding motifs were initially searched across the genome and extracted through 1000 nt of DSS (Additional file 16: Figure S5A-C). Because of their frequent occurrences, all three motifs were again searched through either 500 (Pumilio and Nova) or 200 (PTB) nt of DSS (Table 6). Any transcripts that contained at least four CU runs within this 200-nt DSS were identified as potential PTB targets. Forty-six hundred RNAs were identified with a Pumilio binding motif, 3000 RNAs with a Nova binding motif, and more than 3000 RNAs contain the PTB motif (Table 6, Group 1). Ham et al. [20] demonstrated that the pumpkin PTB protein, RBP50, binds specifically to UUCUCUCUccuUCUU sequences present within a subclass of phloem-mobile, polyadenylated transcripts. On this basis and to ensure more exclusiveness, we screened DSSs of the 31,742 PTB RNA pool for this motif. From this screen, 422 RNAs were identified that contained the RBP50 motif. Included in this list were RNAs encoding a Gag-pol polyprotein (HIV-related), an integrase core domain-containing protein (HIV-related), ethylene response factors, a SET-domain protein, a LOB-domain protein, a tuber-specific element-binding protein, and numerous other TFs, signaling and receptor-like proteins.

**Table 6:** Transcripts with RNA-binding motifs in the 3′ UTR (500 or 200 bp downstream of stop codon)

	Pumilio (500 bp)	Nova (500 bp)	PTB (200 bp)
Group 1			
Transcripts in whole genome (39,028)	4629	3042	31,742
Transcripts with more than 10 reads in phloem-associated tissue (15,167)	2142	1318	13,320
Transcripts with more than 500 reads in phloem-associated tissue (3593)	529	290	3223
Group 2			
PAC abundant & Differentially expressed genes under photoperiod change (2166)	338	168	1943
Group 3			
TFs (sequence-specific DNA binding transcription factor activity) (1090)	190	88	859
TF transcripts with more than 10 reads in PAC (525)	103	39	442
TF transcripts abundant in PAC (163)	28	6	130

After examining the frequency of distribution of these motifs in DSS, only the Pumilio motif demonstrated any significant enrichment. This enrichment occurred in the first 200 nt of DSS. In contrast, the motifs for PTB and Nova were randomly distributed across all DSS examined (Additional file 16: Figure S5A-C). Many of the DSSs analyzed here contained multiple binding motifs. Among the 4629 RNAs containing a Pumilio binding motif, there are 383 with two Pumilio binding motifs, 43 with three, 6 with four and 1 with seven. Among the 3042 RNAs containing multiple Nova binding motifs, there are 176 with two motifs, 10 with three, and 1 with four motifs. There are also different motifs existing in the same DSS (Additional file 16: Figure S5D). Three hundred and twenty DSSs screened contained all three types of RBP motifs. Approximately, 6000 transcripts contained two different RBP binding motifs of Pumilio, Nova or PTB. Unique among the three motifs we searched, Pumilio motif-targeted transcripts were over-represented in 13 gene ontology categories (Additional file 17: Figure S6). Included among these categories were “sequence-specific DNA binding transcription factor activity”, “protein autoubiquitination”, “DNA-dependent regulation of transcription” and “DNA-dependent negative regulation of transcription”. All these functions and biological processes are important in regulating their targets and downstream genes. By its binding to the transcripts of regulatory genes, Pumilio protein indirectly regulates the activity of a wide range of genes.

### Accumulation of RBP targets in PAC

The RBP targets abundantly expressed in PAC are of especial interest. Present in sieve elements, phloem-mobile transcripts associated with RBPs are good candidates for long-distance signals. Among the PAC-abundant transcripts (>500 in petiole-PAC or stem-PAC), 529 contain the Pumilio binding motif, 290 contain the Nova binding motif, and 3223 contain the PTB binding motif (Table 6, Group 1). When considering the photoperiod effect, these numbers are reduced even more at 338, 168, and 1943, respectively (Table 6, Group 2). As an over-represented GO group in Pumilio-targeted transcripts, the 1090 “sequence-specific DNA binding transcription factor activity” related genes in the potato genome (Additional file 17: Figure S6) were also screened for Nova and PTB binding motifs. Among the transcripts of transcription factors in PAC (>10 reads in petiole-PAC or stem-PAC), 103 of them are Pumilio targets, 39 are Nova targets and 442 are PTB targets (red text, Table 6, Group 3; Additional file 18: Table S12). Only 28, 6, and 130 of these transcripts were abundant in PAC (>500 reads), respectively, with Pumilio, Nova and PTB binding motifs.

Several TF families exhibited enrichment of these RBP binding motifs in their DSSs (Table 7). Auxin responsive factors (ARF) and AUX/IAA are two different components in auxin-mediated transcription regulation, as transcription factor and transcriptional repressors, respectively [73]. *AUX*/*IAA* transcripts have been reported to be phloem mobile into roots [12] and eighteen RNAs in this class contain at least one RBP binding motif. One notable RNA is *Auxin response factor 2* (PGSC0003DMG400014179). This RNA accumulates to high levels in both petiole-PAC and stem-PAC and both PTB and Pumilio motifs are present in its DSS. Long-distance movement of transcripts encoding both StBEL1 and StKNOX type TFs has also been previously reported [9, 21]. Only two of the 13 StBEL RNAs, *StBEL22* and −*30*, contained no RNA-binding motifs for the three RBPs we searched. DSS of *STH1* contains all three RNA-binding motifs. All six KN1-like RNAs contain PTB motifs and three of the six are relatively abundant in PACs (Table 7). Other abundant PAC TFs containing RNA motifs include members of the NAC-domain [74] and WRKY [75] families. Two NAC domain RNAs, Nam 9, and two WRKY transcription factors contain both PTB and Pumilio binding motifs in their DSS (Table 7).

**Table 7:** Transcription factors with RNA-binding protein motifs in their downstream sequences

Gene ID	Motif			Annotation	Pet phloem	Stem phloem	LD. mean	SD. mean
PGSC0003DMG400002392	PTB			Auxin_response_factor_7	309	212	954	1209
PGSC0003DMG400003771	PTB			Auxin_response_factor_5	11	149	282	437
PGSC0003DMG400008065	PTB			Auxin_response_factor_4	910	835	2160	2426
PGSC0003DMG400009773	PTB			Auxin_response_factor_19	540	1364	2414	2546
PGSC0003DMG400014179	PTB	PUM		Auxin_response_factor_2	1805	2124	4654	5835
PGSC0003DMG400014452			NOVA	Auxin_response_factor_2	966	346	1795	1904
PGSC0003DMG400015919	PTB			ARF8	826	600	4562	4915
PGSC0003DMG400020711		PUM		Auxin_response_factor_1	750	822	3921	3179
PGSC0003DMG400023345	PTB			ARF_domain_class_transcription_factor	500	206	806	637
PGSC0003DMG401018664	PTB			Auxin_response_factor_8-1	557	508	4626	3659
PGSC0003DMG400000118	PTB			StlAA15 (AtARF9)	2638	2838	2945	3706
PGSC0003DMG400002608			NOVA	StlAA14 (AtlAA18/28)	926	1325	2393	693
PGSC0003DMG400005327	PTB			StlAAlO (AtlAA16)	710	549	958	1483
PGSC0003DMG400006093	PTB			StlAA24 (AtlAA14)	413	178	6503	11,614
PGSC0003DMG400013445	PTB	PUM		StlAA12 (AtlAA3)	0	61	21	20
PGSC0003DMG400020139	PTB	PUM		StlAA2 (AtlAAl)	11	23	382	469
PGSC0003DMG400029339	PTB			StlAA5 (AHAA13)	4	125	36	46
PGSC0003DMG402019457	PTB			Auxin_indole-3-acetic_acid_3(AtlAA16)	534	537	6031	5908
PGSC0003DMG400019635	PTB			BEL11	92	85	4043	5214
PGSC0003DMG400010086	PTB			BEL13	1	162	520	389
PGSC0003DMG400012329	PTB			BEL14	0	0	84	83
PGSC0003DMG400021323	PTB			BEL29	1282	2591	4788	4997
PGSC0003DMG400003750	PTB			BEL31	16	9	382	336
PGSC0003DMG400024267	PTB	PUM		BEL33	464	812	1072	1100
PGSC0003DMG400008057	PTB	PUM		BEL34	199	66	218	399
PGSC0003DMG400005930	PTB			BEL5	2089	1234	3602	3375
PGSC0003DMG400003751		PUM		BEL32	10	47	958	680
PGSC0003DMG400019142		PUM		BEL35	301	453	528	635
PGSC0003DMG400029946		PUM		BEL6	5	60	204	282
PGSC0003DMG400030961				BEL30	9	23	770	769
PGSC0003DMG400022011				BEL22	0	84	57	50
PGSC0003DMG400007887	PTB	PUM		Homeobox_protein_knotted-l-like_LET12	12	13	84	53
PGSC0003DMG400030737	PTB			Homeobox_protein_knotted-l-like_LET12	1132	2984	3147	3270
PGSC0003DMG400004953	PTB			STH20	607	726	186	226
PGSC0003DMG400013493	PTB			POTH1	267	24	77	98
PGSC0003DMG400016711	PTB			STH15 (STM)	4341	3154	192	185
PGSC0003DMG400002769	PTB	PUM	NOVA	STH1	55	61	567	499
PGSC0003DMG400011891	PTB	PUM		NAC_domain_protein	172	25	1368	624
PGSC0003DMG400016896	PTB			NAC_domain_protein	117	266	125	93
PGSC0003DMG400019615	PTB			NAC_domain_protein	112	30	192	179
PGSC0003DMG400032555	PTB			NAC_domain_protein	128	4	112	91
PGSC0003DMG400018435	PTB	PUM		NAC_domain_protein_NAC2	202	104	215	99
PGSC0003DMG400017567	PTB			Nam	461	290	1067	663
PGSC0003DMG400028662	PTB			Nam 2	1248	1999	3026	2292
PGSC0003DMG400031072	PTB			Nam 4	1998	1083	1034	803
PGSC0003DMG400031149	PTB			Nam 7	347	442	809	685
PGSC0003DMG401023373	PTB			Nam 8	47	101	502	471
PGSC0003DMG400031071	PTB	PUM		Nam 9	925	1492	1621	1761
PGSC0003DMG402023373	PTB			Nam 9	395	216	1428	1310
PGSC0003DMG400000064	PTB			WRKY_transcription_factor_23	67	37	98	134
PGSC0003DMG400000211	PTB			WRKY_transcription_factor	520	126	236	164
PGSC0003DMG400001434	PTB			WRKY_transcription_factor-c	9	38	224	166
PGSC0003DMG400005329	PTB	PUM		WRKY_transcription_factor	1579	946	976	918
PGSC0003DMG400005836	PTB			WRKY_transcription_factor-30	42	0	13	10
PGSC0003DMG400009014		PUM		WRKY_transcription_factor_lle-1	157	50	1416	1532
PGSC0003DMG400009530	PTB			WRKY_transcription_factor_3	974	713	580	1000
PGSC0003DMG400015076	PTB	PUM		WRKY_transcription_factor	204	44	184	146
PGSC0003DMG400020432	PTB			WRKY_transcription_factor_5	1	82	98	76
PGSC0003DMG400022063	PTB			WRKY1a_transcription_factor	17	0	38	43
PGSC0003DMG400022143	PTB			WRKY_DNA-binding_protein	1702	1599	2386	1988
PGSC0003DMG400023196	PTB			WRKY_transcription_factor	27	28	1	1
PGSC0003DMG400028520	PTB			WRKY_transcription_factor_1	3534	736	774	559
PGSC0003DMG401031196	PTB			WRKY_transcription_factor_16	571	255	458	186
PGSC0003DMG401033880	PTB			WRKY_transcription_factor_27	36	79	326	360

## Discussion

### Phloem RNA derived from laser capture microdissection

Compared to a genomic study, transcriptome analysis is more informative as it provides a snapshot of processes of physiology and development. RNA levels can be affected by three factors, the rates of transcription, degradation and processes of movement. For phloem-associated cells, the dynamics of transcript levels are even more important, since phloem is the conduit for allocation of photosynthate. In addition to the transcripts that maintain the metabolism and function of phloem, there is also a unique set of non-cell-autonomous mRNAs moving through the phloem as potential long-distance signals. Because of the limitations inherent in the harvest of potato phloem sap, isolation of phloem cells can be readily accomplished by using the well-developed technique of LCM. Early applications of LCM to extract RNA from phloem cells lacked depth and were inefficient. LCM of rice phloem cells yielded only 413 clones that exhibited a high level of redundancy [26]. Refinement of the technique coupled with high-resolution next generation sequencing technology has facilitated expression analysis of select target cells characterized by a very high level of resolution and reproducibility.

In this study, we sequenced the transcriptome of petiole-PAC and stem-PAC using RNA-seq. The raw output is approximately 10^7^ reads for each sample. Out of the approximately 40 k genes in the potato genome, roughly 15 k genes were expressed in PAC of petiole and stem. Our numbers are comparable to the 14,242 and 13,775 active genes identified in the vascular bundles of cucumber and watermelon, respectively [36]. Through statistical analysis, petiole- and stem-PAC exhibited very similar transcriptomes with just slight differences. The genes DE in common between them and the unique genes in each were associated with important GO categories in both signaling and developmental regulation. Approximately 50 DE genes were grouped into signaling-related GO categories, including light, hormone, and flowering related categories. GO categories for binding functions were proportionately over-represented for transcripts abundant in PAC, including both DNA and RNA binding. A propensity for binding and signaling categories for genes expressed in PAC would reflect the dynamic functions of the phloem as a conduit for transporting sucrose and a range of signaling molecules [76].

### Previous expression profiles of phloem

Previous work has established the foundation for RNA profiling of phloem utilizing both LMPC-derived cells and sap harvested from *Arabidopsis* and melon [22, 23]. Transcripts present in sieve elements were identified from melon phloem sap, which readily bleeds from stem cuts [22]. In this study, 1830 unique ESTs were sequenced and mapped to 986 unique transcripts. Using gene functional analysis, 15 % of these genes encoded proteins related to signal transduction. Using these ESTs as a query, 124 potato orthologs were identified from the potato genome. One-hundred and four of them were expressed in either petiole- or stem-PAC. Unfortunately, the profile of the phloem ESTs in melon lacked much depth and expression levels were not verified quantitatively. Using RNA-seq, approximately 10^4^-fold more fragments can be generated and sequenced compared to the EST sequencing approach. This enhanced resolution provides more quantitative sequence information and more insights into the function of the profiled RNAs.

The study on *Arabidopsis* phloem compared the profiles of LMPC-derived phloem tissue and leaf phloem exudate and in this way, provided a hint of the identity of mobile transcripts present in the phloem [23]. Approximately 2400 transcripts were identified in the phloem exudate by microarray, and 90 of them were categorized as functional in signaling pathways. Seventy-six genes in the potato genome were identified as orthologs of these 90 putative mobile transcripts. Seventy of the 76 genes were expressed (>10 reads) in either the petiole- or stem-phloem libraries (Additional file 19: Table S13). Twenty-eight exhibited more than 1000 reads in the petiole-PAC library. These orthologs, including 14-3-3 proteins, MAP kinases, light-related proteins (e.g., one *AUX*/*IAA* RNA), and calcium-responsive signals (Additional file 19: Table S13), represent potential mobile mRNAs in potato. The comparison of profiles in the Deeken et al. study [23] is invaluable but because most plants do not readily yield phloem sap, our current approach using LCM technology coupled with RNA-seq exhibits numerous advantages. It is consistent with previous methods but provides wider applicability, cell specificity, and excellent in-depth sequence resolution. On the downside, the LCM protocol is labor-intensive, may yield small amounts of RNA, and opens the possibility of harvesting cells outside the target tissues. A major advantage of using sap over PACs for examining phloem signaling is the enhanced specificity provided by phloem sap analysis. A PAC profile provides greater overall coverage but less specificity for signal transcripts that move through the sieve element system.

### Accumulation patterns for established phloem-mobile mRNAs

RNAs concentrated in PAC can be specifically located in the sieve element, companion cells or parenchyma cells. Depending on stability and transport dynamics, mobile mRNAs may be concentrated in sieve elements. Through RNA-seq, thousands of abundant phloem transcripts can be profiled, but to verify their mobility requires heterografting experiments with different but related species [6] or RNA movement assays [77]. Another option is to generate stably transformed plants that express the test RNA with a non-plant sequence tag and heterograft with wild type plants [9]. All three approaches are labor-intensive and time consuming. The *in silico* analysis approach for identifying conserved motifs in 3′ UTRs presented in this study (Tables 6 and 7) has the clear potential to more efficiently predict candidate transcripts for long-distance mobility.

Non-cell-autonomous mRNAs may move into the sieve element system through plasmodesmata connecting companion cells to sieve elements [78]. In this model, any RNAs from the leaf, transported long distance, may be detected in both petiole- and stem-PAC. As discussed previously, there are hundreds of full-length mRNAs present in phloem sap but only a few of these have been confirmed to move and even fewer are associated with a phenotype [15]. This short list includes *LeT6* in tomato, *GAI* in pumpkin, tomato, and *Arabidopsis*, *IAA18*/*28* in *Arabidopsis*, and *POTH1* and *StBEL5* of potato. The potato orthologs of these mobile RNAs along with *StBEL5* and *POTH1* are detected in the phloem-associated cells of both petiole and stem in relatively abundant levels (Table 8). Because there are reports of the transport of FT mRNA [11, 79], the FT/SP6A orthologs of potato are also included. The complete absence of any accumulation of transcripts for *StFT* genes suggests that these RNAs are not phloem-mobile. Evidence indicates that SP6A is translated in leaves and moves through the phloem to underground stolons in protein form [14]. *STH15* and *StBEL5* exhibited the greatest levels of accumulation in PAC (Table 8). Of course, abundance levels of any specific RNA would be determined by the rate of transcription, the stability of the RNA, and the degree of its mobilization.

**Table 8:** Accumulation of known mobile mRNAs in stem and petiole PACs

Annotation	Gene ID	Petiole phloem	Stem phloem	Function	Citation
STH15 (STM, LeT6)	PGSC0003DMG400016711	4341	3154	Leaf morphology	Kim et al., [10]; Ham et al., [20]
DELLA protein GAI	PGSC0003DMG400015692	531	422	Leaf morphology	Haywood et al., [8]
StBEL5	PGSC0003DMG400005930	2089	1234	Tuber and root growth	Banerjee et al., [9]; Lin et al., [19]
IAA18/28	PGSC0003DMG400002608	926	1325	Root growth	Notaguchi et al., [12]
POTH1	PGSC0003DMG400013493	267	24	Vegetative growth	Mahajan et al., [21]
FT	PGSC0003DMG400016179	0	0	Flowering	Li et al., [11]
SP6A	PGSC0003DMG400023365	0	0	Tuberization	Navarro et al., [14]

*GA INSENSITIVE* (*GAI*) is exceptional in that long-distance movement of its mRNA has been established in several plant species including cucumber, tomato, pumpkin [8, 20], apple [80], and *Arabidopsis* [81]. It was the first mobile RNA identified and CU-rich sequences in its transcript facilitate binding to CmRBP50, a PTB protein of pumpkin. Accumulation of *AtGAI* across a graft union can affect leaf architecture [8]. *IAA18*/*28* was verified to cross graft unions and move into root tips to regulate root architecture [12]. *STH15* is the ortholog of *LeT6* of tomato and *STM* of *Arabidopsis*. Mobility assays of *LeT6* confirmed upward movement of is transcript associated with a leaf phenotype in tomato [10]. Both *POTH1* and *StBEL5* have been associated with tuber development [82, 83]. Movement of *StBEL5* is induced by short days and regulated by its UTRs. The UTRs of *POTH1*, *StBEL5*, and *StGAI* interacted with a potato PTB protein [21]. Because several of these mobile RNAs interact with the same RBP, it is conceivable that multiple RNAs are transported in the same RNP complex. For example, the mobile RNA/RBP50 complex of pumpkin contained six RNAs including *CmGAI* and *CmSTM*. All six of these RNAs contained CU-rich PTB motifs. These CU-rich motifs were also observed in the UTRs of *StBEL5*, *POTH1*, *StGAI*, and *STH1*5.

### The role of RNA-binding proteins

RBPs mediate numerous aspects of RNA metabolism including mRNA capping, rate of degradation, translation, localization and transport. For long-distance mobilization of mRNAs, RBPs associated with them are especially important in stabilizing and localizing the mRNAs, while repressing translation during the process. Our analysis revealed numerous transcripts encoding RBPs in both petiole- and stem-PAC (Additional file 6: Table S5) and it is very likely that a subgroup of these are functional in the execution of mRNA transport via the sieve elements. The glycine-rich protein 7 (GRP7) was the most abundant RBP in our libraries. Seven KH domain proteins (including Nova), four Pumilio proteins, and all six potato PTBs were identified (Table 5).

Surprisingly, one of the most abundant RBPs was Pumilio1. Pumilio proteins are post-transcriptional regulators containing Puf domains (Pumilo and FBF) that recognize RNA sequences present in the 3′ UTR of target RNAs. Pumilio functions in cytoplasmic de-adenylation, translational repression, RNA localization and decay, maintenance of germline stem cell identity, translation initiation, and rRNA processing and ribosome biogenesis [68]. Puf proteins repress translation of target RNAs during establishment of polarity in the developing embryo of *Drosophila* and during the localization of *Ash1* mRNA to the distal tip of the budding cell [84, 85]. They bind to RNAs at a motif containing a conserved UGUR (where R is a purine). Despite its importance, only a scarcity of information is available on the function of these RBPs in plants [59, 61]. Whereas the Pumilio protein, APUM23, functions in polarity formation in *Arabidopsis* [61], the role of any Puf proteins in vascular biology is completely unknown.

As previously mentioned, a PTB protein was identified as the core protein in a mobile RNA/protein complex in the phloem of pumpkin [20]. RBP50 has two orthologs in the potato genome, designated StPTB1 and StPTB6. The PTB family of RNA-binding proteins are functional in a wide range of posttranscriptional processes including RNA stability [86], splicing regulation [87], localization [88], translation control [89], and long-distance transport [20]. There are two subfamilies of plant PTBs. One is represented by StPTB1, StPTB6, CmRBP50, and AtPTB3 and these are speculated to be involved in long-distance movement. A second subfamily of PTB proteins, represented by AtPTB1 and −2 and the StPTB7 types, function in alternative splicing [57, 90]. The KH-domain protein, Nova, binds to both StPTB1 and −6 [56] and a Nova ortholog was identified in pumpkin phloem sap [2]. Alba was included because it interacts with the mobile RNA *POTH1* [21]. Identification of RBPs and their target RNAs in potato PAC provides a valuable experimental framework for testing interactions between proteins and RNAs that may be functional in long-distance signaling processes. For example, screening for binding elements in RNA sequences comparable to the approach implemented for the PTBs, Nova, and Pumilio in this study could be readily performed for any RBP of interest.

## Conclusions

Our results confirm that the combination of laser capture microdissection and RNA-seq provides an invaluable and in-depth approach to the study of phloem biology and a comprehensive picture of the mechanisms associated with long-distance signaling and transport. Out of the roughly 39 k genes in the potato genome, approximately 15 k were expressed in PAC of petiole and stem, numbers that are comparable to the number of genes identified in the vascular bundles of cucumber and watermelon [36]. Our GO analysis indicates that signaling and binding processes are important biological activities associated with phloem cells. The high proportion of RBPs in the expression profiles and the high percentage of transcripts containing binding motifs for three prominent RBPs in their downstream sequences suggest an important role for RNA binding in vascular tissue. The results of this study illustrate the potential of RNA profiling for providing insights into long-distance transport processes mediated by environmental cues that are associated with the sieve element system.

## Methods

### Plant material

All RNA samples were from the photoperiod-responsive genotype *S. tuberosum* ssp. *andigena*. This subspecies only tuberizes under SDs. Plants were propagated in vitro on MS media to root and cultured for 4 weeks before moving to soil. The plants were first transplanted to 7.5-cm square pots and covered with plastic to maintain humidity for a week. After 10–14 days, plants were transferred to 15-cm round pots. The plants were maintained in a growth chamber under a long-day photoperiod (16 h light at 22 °C, 8 h dark at 18 °C, with a fluence rate of 280 μmol m^−2^ s^−1^) for 4 weeks before being grown under long-day (16 h light at 22 °C, 8 h dark at 18 °C) or short-day (8 h light at 22 °C, 16 h dark at 18 °C) conditions for 10 more days.

### Sample preparation and laser capture microdissection

Two to three-millimeter tissue segments of *Solanum tuberosum* ssp. *andigena* plants grown under short-days were excised from the central regions of lower stem internodes (4 to 6 cm above the soil line) or petioles from fully emerged leaves near the top of the plant. At harvest, stem or petiole segments were immersed in at least 10-fold volumes (*v*/*v*) of cold fixative (75 % ethanol and 25 % acetic acid) contained in glass vials on ice. Samples were evacuated (0.067 MPa) for 30 min on ice and then fixed 6 h (petioles) or 24 h (stems) in a 4 °C cold room. Tissue segments were transferred to an excess volume of 75 % ethanol (*v*/*v*) at 4 °C for 1 h. The process was repeated once to remove excess fixative. Tissues were dehydrated and paraffin-embedded following protocols of Cai and Lashbrook [91]. Multiple tissue segments were arranged vertically in each embedding mold. Metal embedding molds were sequentially washed with xylol and ethanol prior to air-drying and use. Embedded tissues were stored at −20 °C in sealed containers prior to paraffin sectioning. Tissue cross sections (8 μm thick) were cut on a rotary microtome (AO Spencer 820 Microtome; American Optical) using Leica blades. Paraffin sections were stretched for 1 min onto Probe-on Plus slides (Fisher Scientific) containing 5 mM EDTA in DEPC-treated water, pH 8, at 42 °C. Slides were air-dried at RT for up to 5 h before laser microdissection coupled to laser pressure catapulting (LMPC). Immediately before LMPC, slides were deparaffinized twice in xylol for 15 min each and air dried at RT. One-half ml LMPC tubes with clear, non-adhesive caps (Zeiss, Hamburg, Germany) were disinfected prior to use by submerging in chloroform followed by air-drying. For microdissection, the PALM® Laser Microbeam instrument (Bernried, Germany) was employed. A pulsed UV nitrogen laser beam is projected through the objective lens to a narrow diameter (<1.0 μm) that ablates the target without heating adjacent material. Cells were selected using the graphic tools of the PALM RoboSoftware. Laser pressure catapulting (LPC), with a high photonic pressure force, was used to capture the target phloem cells into the lid of a LPC-microfuge tube containing 25 μl extraction buffer from a Picopure RNA kit (Arcturus Engineering, Mountain View, CA, USA). For each sample an area of approximately 1.5 × 10^6^ μm^2^ comprised of approximately 5000 cells was collected. To minimize degradation, total harvest time was restricted to 1 h per sample. After cell collection, tubes were inverted and the cap end was vortexed in several short spurts to release cells. Contents of the upright tube were pulsed in a microfuge, incubated for 30 min in a water bath at 42 °C, centrifuged at 800 × g for 2 min and stored at −80 °C until RNA isolation.

### RNA isolation, library preparation and sequencing

RNA was isolated from microdissected cells with the PicoPure RNA isolation kit (Arcturus Engineering, Mountain View, CA, USA), incorporating an on-column treatment step with RNase-Free DNase (Qiagen, Valencia, CA, USA, Cat #79254). Finally, RNA was quantified with an Agilent 2100 Bioanalyzer using reagents from the manufacturer’s RNA 6000 Pico kit and then stored at −80 °C. Purified RNAs were amplified using Ovation® RNA-Seq system (NuGEN). cDNA libraries were prepared using 2.0 μg of amplified cDNAs and sequenced at the DNA Facility, Iowa State University. For the LD/SD petiole experiment, 2 to 3 cm petiole sections near the junction of the petiole and stem were excised and harvested for both photoperiod conditions. Total RNA was extracted from petioles of long-day or short-day grown *S. tuberosum* ssp. *andigena* using RNeasy mini kit (Qiagen). After validation of the quality of RNAs using the 2100 Bioanalyzer, approximately 3.0 μg of total RNA were used for library preparation and sequenced using the HiSeq2500 (Illumina) at the DNA Facility, Iowa State University. The set of LCM isolated samples was sequenced with either Genomic DNA/cDNA/BAC GA II 75-Cycle or mRNA-Seq HiSEQ High Output 100-Cycle. The set of whole petiole samples was sequenced with mRNA-Seq HiSEQ High Output 100-Cycle P.E.

### Processing of reads

All the reads were processed as output in fastq format. These reads were aligned to the potato genome (PGSC_DM_v4.03_pseudomolecules.fasta & PGSC_DM_V403_genes.gff) from the potato genome database http://solanaceae.plantbiology.msu.edu/pgsc_download.shtml) with GMAP and GSNAP (http://research-pub.gene.com/gmap/). Parameters were set as default. The number of concordant unique reads in each library was counted with HTseq (http://www-huber.embl.de/users/anders/HTSeq/doc/overview.html). The disparity of reads that were mapped to the genome in a concordant and unique manner between the LCM-PACs (25 to 47 %, Additional file 1: Table S1) and the whole petiole (~94 %, Additional file 9: Table S8) samples is most likely explained by the fact that the LCM-derived RNA was collected in picogram amounts and subsequently amplified, whereas the whole petiole RNA was not [92]. It would appear that amplification produced reads that map to multiple locations disproportionately over the uniquely mapped reads. Because samples were only compared between the same tissue types (stem PAC vs. pet PAC or LD pet vs. SD pet), no sample bias was introduced.

### Statistical analysis

All the libraries were normalized with the 0.75 quantile to eliminate the difference caused by sample scale and sequencing depth. The LCM-derived libraries were sequenced with two different sequencing methods, paired-end and single-end sequencing. Both the difference coming from petiole vs. stem organs and the difference coming from different sequencing methods were considered in the Generalized Linear Model. With the R package “QuasiSeq” [93] (http://cran.rproject.org/web/packages/ QuasiSeq/index.html), quasi-negative binomial deviances of each gene were computed, and the normalized count data was fitted with a quasi-likelihood model. DE genes were selected with adjusted *p*-values less than 0.2. The *p*-value was adjusted with the method from Nettleton et al. [94]. Effect from single-end sequencing method is removed based on the derived coefficient. LD- and SD-petiole samples were sequenced with a multiplexing tag in the same lane, so the photoperiod effect is the only effect to be analyzed in comparison. The count of each gene in each sample type is the mean value of the normalized reads of the three or four replicates.

### GO analysis and GOSeq

Gene ontology categories of all the genes in potato were obtained from the GO database using Blast2GO (http://www.blast2go.com/b2glaunch/start-blast2go), with parameters of 20 hits and an e-value of 10e^−6^ (Additional file 20: Table S14). This analysis was performed on the iPlant platform. Gene ontology analysis was performed with GOseq [64] to identify over-represented GO terms in the DE genes. A probability weighting function (PWF) was generated based on transcript length and was applied to eliminate the bias arising from this parameter. The transcript length was obtained from the longest transcript sequence available from the potato genome database (PGSC_DM_v3.4_transcript-update_representative.fasta) for each gene. When the number of over-represented GO terms was too large to visualize, GO terms were reduced with GOslim (http://agbase.msstate.edu/cgi-bin/tools/goslimviewer_select.pl).

### Motif search

For the motif search, the potato genome (V4.03) and annotations from the Potato Genome Sequencing Consortium [62, 95] were utilized. The position weight matrices for Nova and Pumilio motifs were obtained from Jiang et al. [67]. We searched the entire potato genome for the presence of these motifs using MEME suite [71]. Because the exact length of the 3′ UTR is currently unavailable, we chose an arbitrary fixed length of 1000 nt for the UTR. For each gene, the 1000 bp region downstream from the end of the coding sequence was considered as its 3′ UTR in this analysis. Finally, using the ‘intersectBed’ tool (BEDTools v2.18.2) [72], we extracted the genes containing Nova or Pumilio motifs in the designated 3′ UTRs. The position of motifs was also identified using the ‘intersectBed’ tool.

### Real time RT-PCR

RNA preparation and RT-qPCR were performed as previously described [48]. Primers are listed in Additional file 21: Table S15.

## Availability of supporting data

Sequence reads of the LCM RNA-seq and SD/LD petiole RNA-seq have been deposited in NCBI-SRA in a FASTQ file. DOI: http://www.ncbi.nlm.nih.gov/bioproject/PRJNA290800

## Additional files

Additional file 1: Table S1. Summary of RNA-seq output of LCM collected PAC samples. (XLSX 43 kb) Additional file 2: Figure S1. Distribution of *p*-value and *q*-value of gene expression difference between petiole phloem-associated cells and stem PACs. (PPTX 66 kb) Additional file 3: Table S2. Complete RNA-seq profile. (XLSX 3811 kb) Additional file 4: Table S3. Differentially expressed genes between petiole and stem phloem-associated cells. (XLSX 192 kb) Additional file 5: Table S4. Expression of genes in pet-PAC and stem-PAC with select GO terms. (XLSX 331 kb) Additional file 6: Table S5. Expression level of RNA-binding proteins. (XLSX 78 kb) Additional file 7: Table S6. RNA profiles of select RNA-binding proteins. (XLSX 106 kb) Additional file 8: Table S7. Abundant transcripts in PAC. (XLSX 324 kb) Additional file 9: Table S8. RNA-seq output of petiole samples under photoperiod treatments. (XLSX 45 kb) Additional file 10: Figure S2. Distribution of *p*-value and *q*-value of photoperiod effect on petiole transcriptome. (PPTX 66 kb) Additional file 11: Table S9. Differentially expressed photoperiod genes. (XLSX 1172 kb) Additional file 12: Table S10. Abundant transcripts significantly up-regulated under long days. (XLSX 77 kb) Additional file 13: Table S11. Abundant transcripts significantly up-regulated under short days. (XLSX 59 kb) Additional file 14: Figure S3. Top twenty over-represented GO terms for molecular functions in differentially expressed photoperiod genes. (PPTX 67 kb) Additional file 15: Figure S4. Top twenty over-represented GO terms for biological processes in differentially expressed photoperiod genes. (PPTX 69 kb) Additional file 16: Figure S5. Distribution of RNA-binding protein motifs in potato transcriptome. (PPTX 105 kb) Additional file 17: Figure S6. RNAs containing the Pumilio binding motif are over-represented in thirteen GO categories. (PPTX 70 kb) Additional file 18: Table S12. RNA-seq of RNA-binding proteins and select TFs. (XLSX 54 kb) Additional file 19: Table S13. Comparison to Arabidopsis phloem transcripts. (XLSX 42 kb) Additional file 20: Table S14. Source of GO information for entire potato genome. (TXT 17703 kb) Additional file 21: Table S15. Primers for real time RT-PCR. (XLSX 39 kb)

## Abbreviations

CU: Cytosine/uracil
DSS: Downstream sequence
FD: Flowering Locus D
FT: Flowering Locus T
GO: Gene ontology
LCM: Laser capture microdissection
LD: Long day
PAC: Phloem-associated cells
PTB: Polypyrimidine tract-binding protein
RBP: RNA-binding protein
SD: Short day
UTR: Untranslated region
