# Supplementary material for: Transcriptional analysis of phloem-associated cells of potato
Source: BMC Genomics. 2015 Sep 3;16(1):665. doi: 10.1186/s12864-015-1844-2 (PMC4558636; doi:10.1186/s12864-015-1844-2)
Supplement: Additional file 2: Figure S1. — Distribution of p-value and q-value of gene expression difference between petiole phloem-associated cells and stem PACs. (PPTX 66 kb) [file 12864_2015_1844_MOESM2_ESM.pptx]

## Slide 1
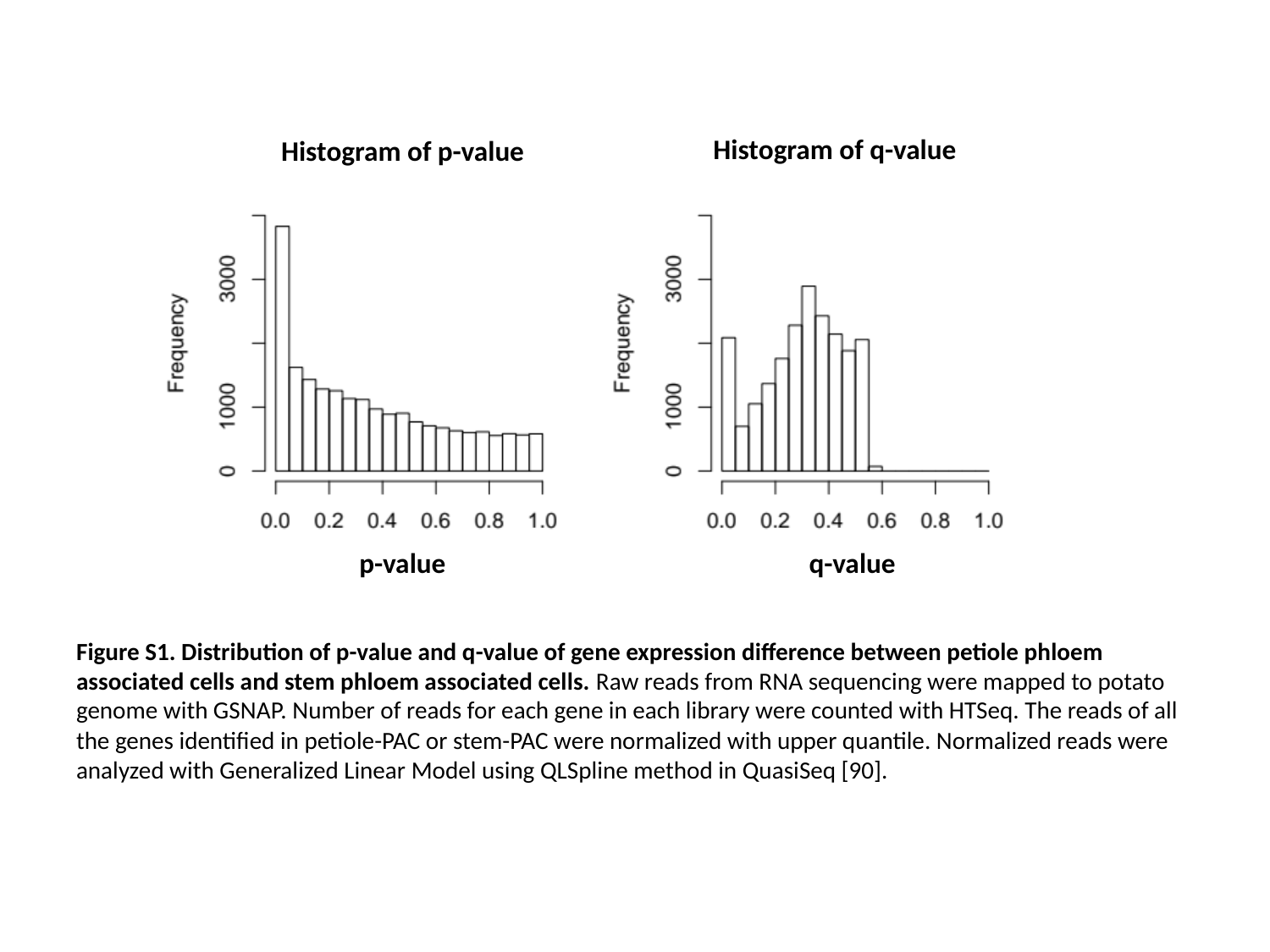

Histogram of q-value
Histogram of p-value
p-value
q-value
# Figure S1. Distribution of p-value and q-value of gene expression difference between petiole phloem associated cells and stem phloem associated cells. Raw reads from RNA sequencing were mapped to potato genome with GSNAP. Number of reads for each gene in each library were counted with HTSeq. The reads of all the genes identified in petiole-PAC or stem-PAC were normalized with upper quantile. Normalized reads were analyzed with Generalized Linear Model using QLSpline method in QuasiSeq [90].
