# Supplementary material for: Transcriptional analysis of phloem-associated cells of potato
Source: BMC Genomics. 2015 Sep 3;16(1):665. doi: 10.1186/s12864-015-1844-2 (PMC4558636; doi:10.1186/s12864-015-1844-2)
Supplement: Additional file 10: Figure S2. — Distribution of p-value and q-value of photoperiod effect on petiole transcriptome. (PPTX 66 kb) [file 12864_2015_1844_MOESM10_ESM.pptx]

## Slide 1
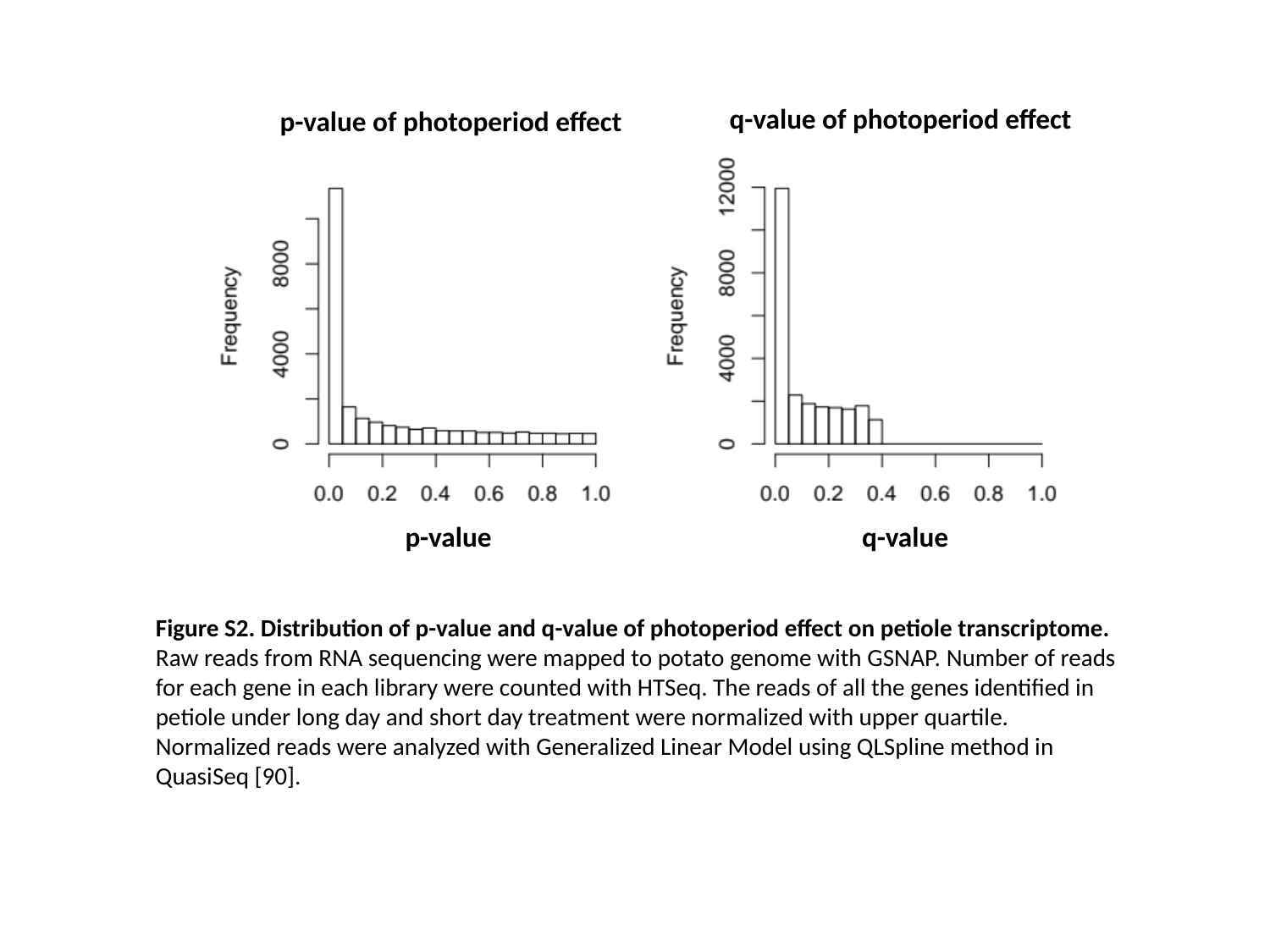

q-value of photoperiod effect
p-value of photoperiod effect
p-value
q-value
Figure S2. Distribution of p-value and q-value of photoperiod effect on petiole transcriptome. Raw reads from RNA sequencing were mapped to potato genome with GSNAP. Number of reads for each gene in each library were counted with HTSeq. The reads of all the genes identified in petiole under long day and short day treatment were normalized with upper quartile. Normalized reads were analyzed with Generalized Linear Model using QLSpline method in QuasiSeq [90].
