# Supplementary material for: Transcriptional analysis of phloem-associated cells of potato
Source: BMC Genomics. 2015 Sep 3;16(1):665. doi: 10.1186/s12864-015-1844-2 (PMC4558636; doi:10.1186/s12864-015-1844-2)
Supplement: Additional file 14: Figure S3. — Top twenty over-represented GO terms for molecular functions in differentially expressed photoperiod genes. (PPTX 67 kb) [file 12864_2015_1844_MOESM14_ESM.pptx]

## Slide 1
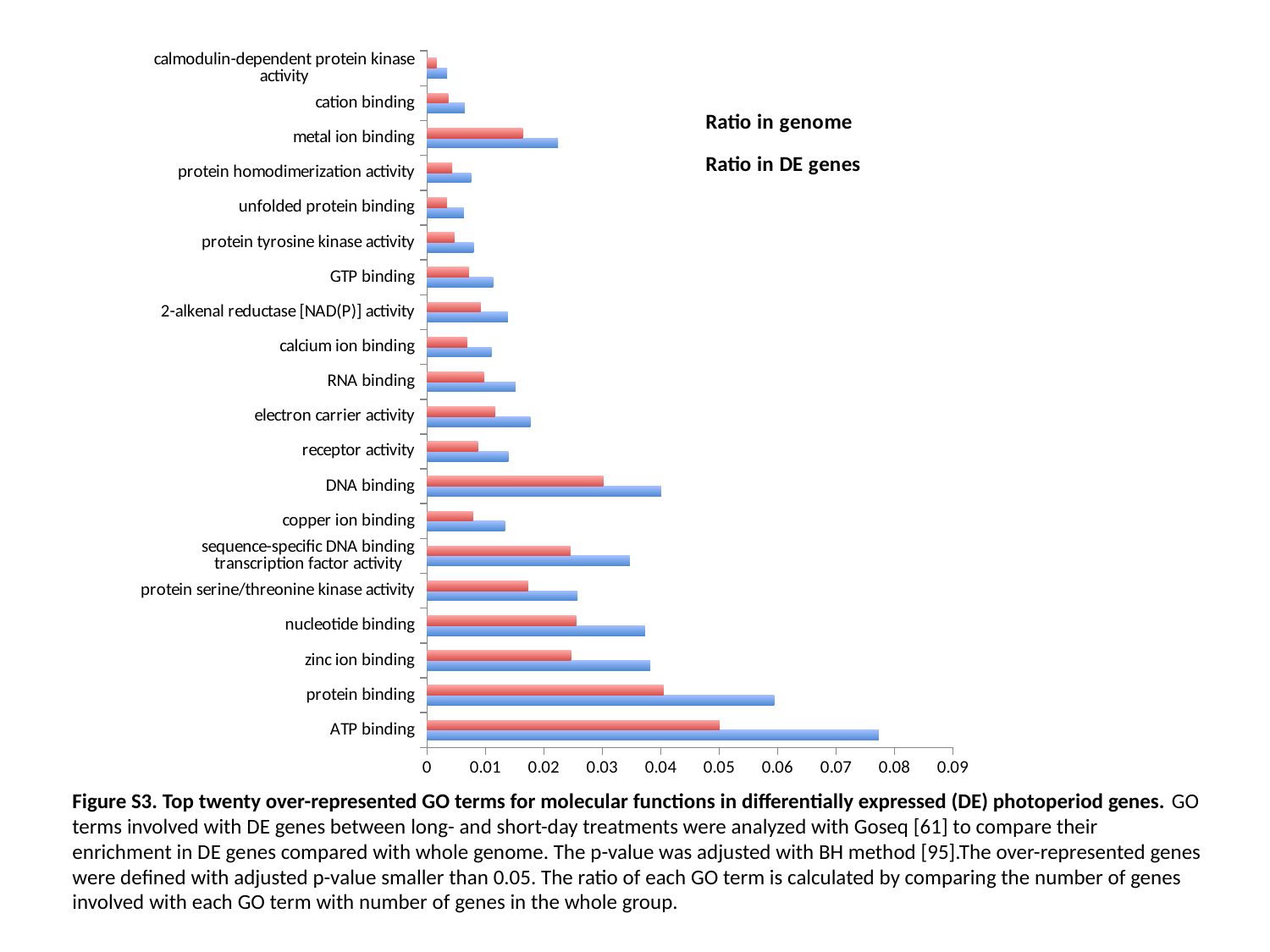

### Chart
| Category | | |
|---|---|---|
| ATP binding | 0.077276908923643 | 0.0500666188377575 |
| protein binding | 0.059379443004098 | 0.0404325099928257 |
| zinc ion binding | 0.0382202893702434 | 0.024623347340371 |
| nucleotide binding | 0.0373003261687714 | 0.0254945167572 |
| protein serine/threonine kinase activity | 0.0257589696412143 | 0.0172440299272317 |
| sequence-specific DNA binding transcription factor activity | 0.0347077026009869 | 0.0244952341908373 |
| copper ion binding | 0.0132976499121853 | 0.00784052475146049 |
| DNA binding | 0.0400602157731872 | 0.0301578354002255 |
| receptor activity | 0.0138830810403947 | 0.00866044890847596 |
| electron carrier activity | 0.0176465668645981 | 0.0116070513477503 |
| RNA binding | 0.0151375763151292 | 0.00971097673465204 |
| calcium ion binding | 0.0110395584176633 | 0.00681561955519114 |
| 2-alkenal reductase [NAD(P)] activity | 0.0137994480220791 | 0.0091472788767039 |
| GTP binding | 0.0112904574726102 | 0.00717433637388541 |
| protein tyrosine kinase activity | 0.00794513673998494 | 0.00466331864302552 |
| unfolded protein binding | 0.00627247637367232 | 0.00338218714768884 |
| protein homodimerization activity | 0.00752697164840679 | 0.00427897919442451 |
| metal ion binding | 0.0224136489085891 | 0.0163728605104028 |
| cation binding | 0.00643974241030359 | 0.00361279081684944 |
| calmodulin-dependent protein kinase activity | 0.00342895375094087 | 0.00161422568412422 |# Figure S3. Top twenty over-represented GO terms for molecular functions in differentially expressed (DE) photoperiod genes. GO terms involved with DE genes between long- and short-day treatments were analyzed with Goseq [61] to compare their enrichment in DE genes compared with whole genome. The p-value was adjusted with BH method [95].The over-represented genes were defined with adjusted p-value smaller than 0.05. The ratio of each GO term is calculated by comparing the number of genes involved with each GO term with number of genes in the whole group.
