# Supplementary material for: Transcriptional analysis of phloem-associated cells of potato
Source: BMC Genomics. 2015 Sep 3;16(1):665. doi: 10.1186/s12864-015-1844-2 (PMC4558636; doi:10.1186/s12864-015-1844-2)
Supplement: Additional file 15: Figure S4. — Top twenty over-represented GO terms for biological processes in differentially expressed photoperiod genes. (PPTX 69 kb) [file 12864_2015_1844_MOESM15_ESM.pptx]

## Slide 1
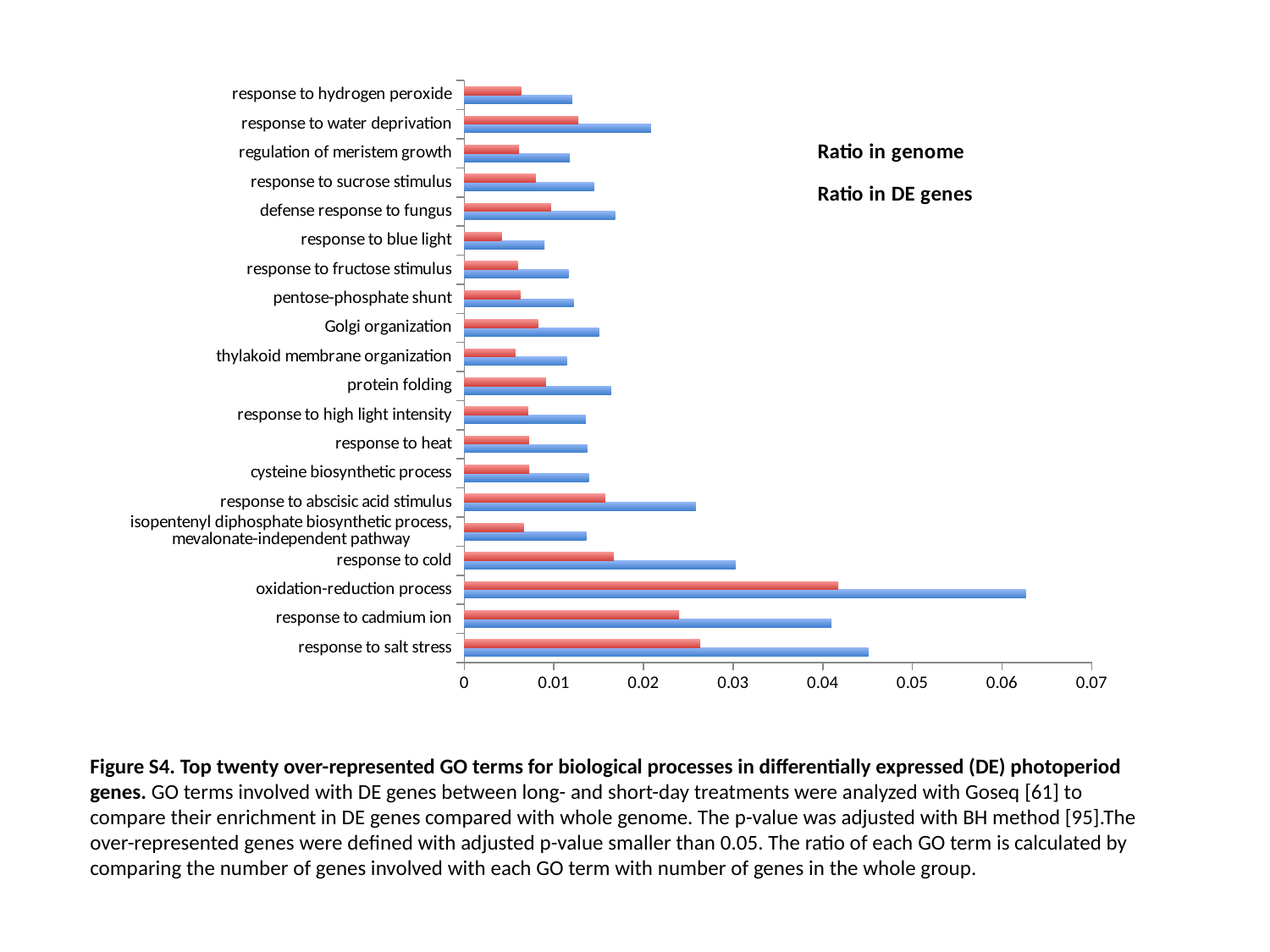

### Chart
| Category | | |
|---|---|---|
| response to salt stress | 0.0450781968721251 | 0.0263400635441222 |
| response to cadmium ion | 0.0409801789746592 | 0.0239827815927027 |
| oxidation-reduction process | 0.0626411307184076 | 0.0417392641180691 |
| response to cold | 0.0302751526302584 | 0.0166803320692836 |
| isopentenyl diphosphate biosynthetic process, mevalonate-independent pathway | 0.0136321819854479 | 0.00663626114584401 |
| response to abscisic acid stimulus | 0.02584260265953 | 0.0157322947627344 |
| cysteine biosynthetic process | 0.0138830810403947 | 0.00725120426360561 |
| response to heat | 0.0137158150037635 | 0.00722558163369888 |
| response to high light intensity | 0.0135485489671322 | 0.00712309111407195 |
| protein folding | 0.0163920715898637 | 0.00912165624679717 |
| thylakoid membrane organization | 0.0114577235092414 | 0.0057138464692016 |
| Golgi organization | 0.0150539432968136 | 0.00822486420006149 |
| pentose-phosphate shunt | 0.0122104206740821 | 0.006251921697243 |
| response to fructose stimulus | 0.0116249895458727 | 0.00597007276826893 |
| response to blue light | 0.00894873295977252 | 0.00415086604489085 |
| defense response to fungus | 0.0168102366814418 | 0.00968535410474531 |
| response to sucrose stimulus | 0.0144685121686042 | 0.00796863790099416 |
| regulation of meristem growth | 0.011792255582504 | 0.00612380854770933 |
| response to water deprivation | 0.0208246215605921 | 0.0127344470636466 |
| response to hydrogen peroxide | 0.0120431546374509 | 0.0063287895869632 |Figure S4. Top twenty over-represented GO terms for biological processes in differentially expressed (DE) photoperiod genes. GO terms involved with DE genes between long- and short-day treatments were analyzed with Goseq [61] to compare their enrichment in DE genes compared with whole genome. The p-value was adjusted with BH method [95].The over-represented genes were defined with adjusted p-value smaller than 0.05. The ratio of each GO term is calculated by comparing the number of genes involved with each GO term with number of genes in the whole group.
