# Supplementary material for: Transcriptional analysis of phloem-associated cells of potato
Source: BMC Genomics. 2015 Sep 3;16(1):665. doi: 10.1186/s12864-015-1844-2 (PMC4558636; doi:10.1186/s12864-015-1844-2)
Supplement: Additional file 16: Figure S5. — Distribution of RNA-binding protein motifs in potato transcriptome. (PPTX 105 kb) [file 12864_2015_1844_MOESM16_ESM.pptx]

## Slide 1
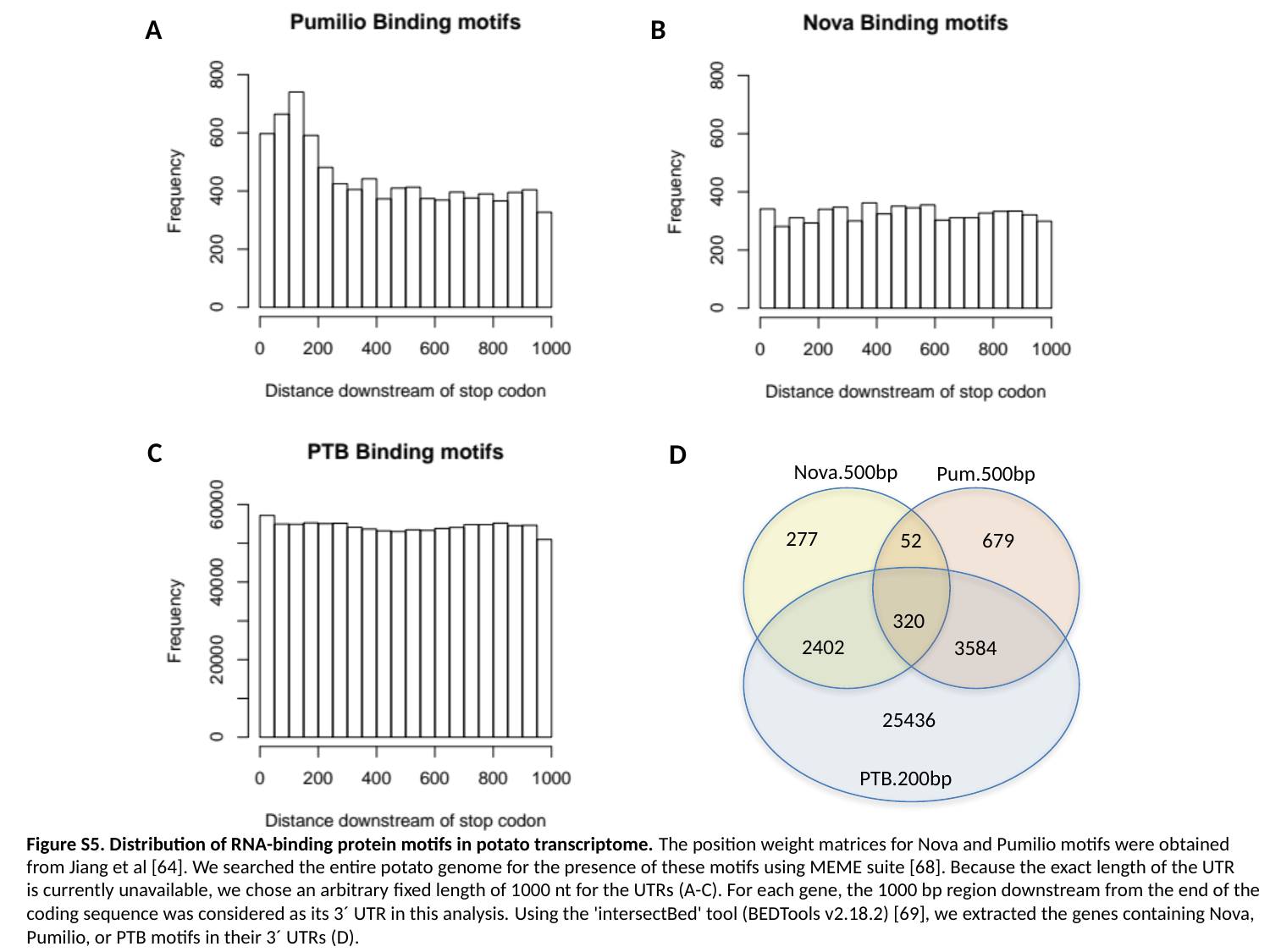

A
B
C
D
Nova.500bp
Pum.500bp
277
52
679
320
2402
3584
25436
PTB.200bp
Figure S5. Distribution of RNA-binding protein motifs in potato transcriptome. The position weight matrices for Nova and Pumilio motifs were obtained from Jiang et al [64]. We searched the entire potato genome for the presence of these motifs using MEME suite [68]. Because the exact length of the UTR is currently unavailable, we chose an arbitrary fixed length of 1000 nt for the UTRs (A-C). For each gene, the 1000 bp region downstream from the end of the coding sequence was considered as its 3´ UTR in this analysis. Using the 'intersectBed' tool (BEDTools v2.18.2) [69], we extracted the genes containing Nova, Pumilio, or PTB motifs in their 3´ UTRs (D).
