# Supplementary material for: Transcriptional analysis of phloem-associated cells of potato
Source: BMC Genomics. 2015 Sep 3;16(1):665. doi: 10.1186/s12864-015-1844-2 (PMC4558636; doi:10.1186/s12864-015-1844-2)
Supplement: Additional file 17: Figure S6. — RNAs containing the Pumilio binding motif are over-represented in thirteen GO categories. (PPTX 70 kb) [file 12864_2015_1844_MOESM17_ESM.pptx]

## Slide 1
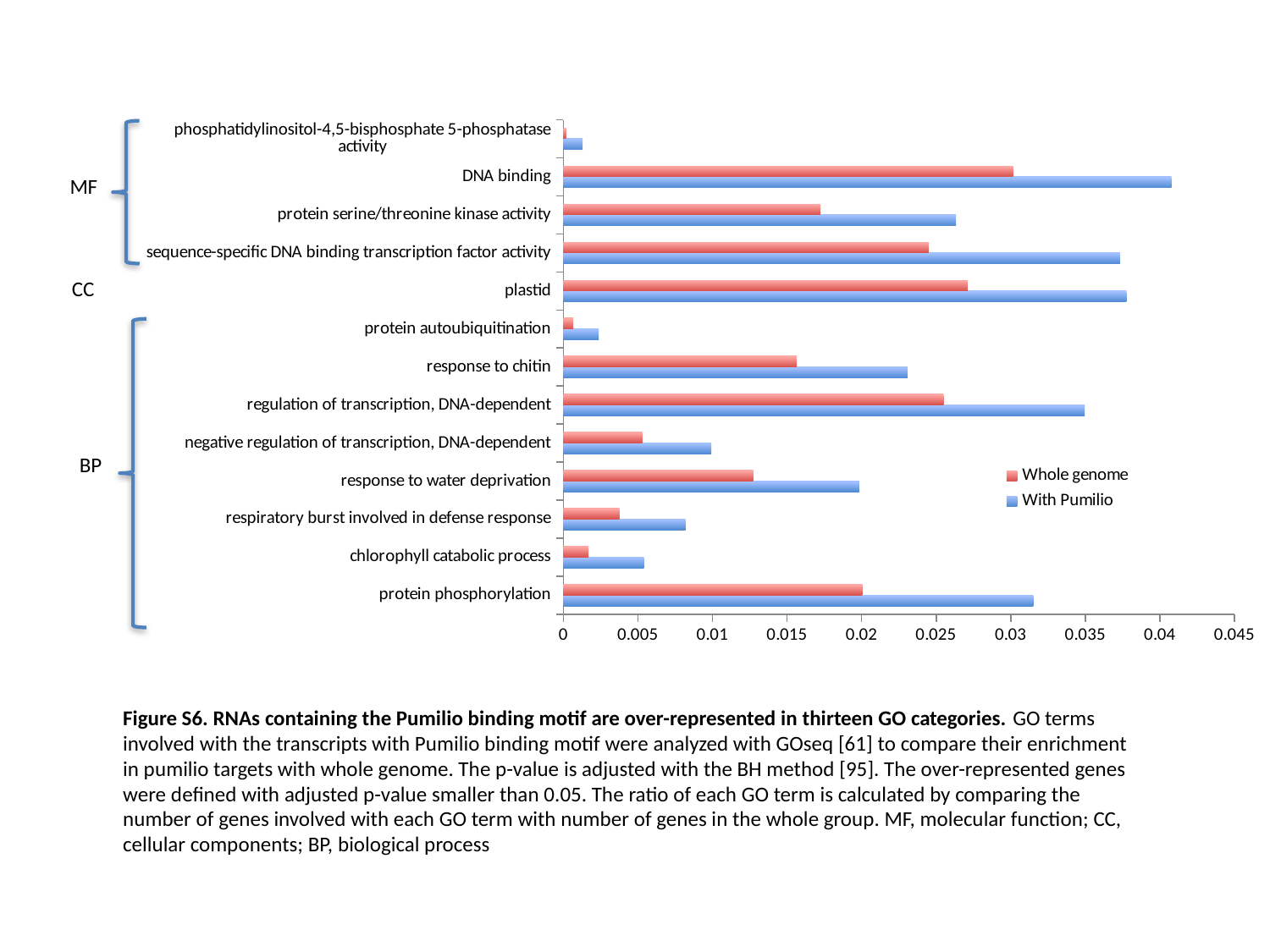

### Chart
| Category | | |
|---|---|---|
| protein phosphorylation | 0.0314994606256742 | 0.0200368965870657 |
| chlorophyll catabolic process | 0.00539374325782093 | 0.00169109357384442 |
| respiratory burst involved in defense response | 0.00819848975188781 | 0.00376652659628984 |
| response to water deprivation | 0.019848975188781 | 0.0127344470636466 |
| negative regulation of transcription, DNA-dependent | 0.0099244875943905 | 0.00530388439069386 |
| regulation of transcription, DNA-dependent | 0.0349514563106796 | 0.0254945167572 |
| response to chitin | 0.0230852211434736 | 0.0156554268730142 |
| protein autoubiquitination | 0.00237324703344121 | 0.000666188377575074 |
| plastid | 0.0377562028047465 | 0.0271087424413242 |
| sequence-specific DNA binding transcription factor activity | 0.0373247033441208 | 0.0244952341908373 |
| protein serine/threonine kinase activity | 0.0263214670981661 | 0.0172440299272317 |
| DNA binding | 0.0407766990291262 | 0.0301578354002255 |
| phosphatidylinositol-4,5-bisphosphate 5-phosphatase activity | 0.00129449838187702 | 0.000179358409347135 |
MF
CC
BP
Figure S6. RNAs containing the Pumilio binding motif are over-represented in thirteen GO categories. GO terms involved with the transcripts with Pumilio binding motif were analyzed with GOseq [61] to compare their enrichment in pumilio targets with whole genome. The p-value is adjusted with the BH method [95]. The over-represented genes were defined with adjusted p-value smaller than 0.05. The ratio of each GO term is calculated by comparing the number of genes involved with each GO term with number of genes in the whole group. MF, molecular function; CC, cellular components; BP, biological process
